# Immuno-informatics voyage through molecular mimicry of Heat Shock Proteins: Potential IBD immunopathogenesis

**DOI:** 10.1371/journal.pone.0333618

**Published:** 2025-10-16

**Authors:** Mahsa Alem, Seyyed Meysam Abtahi Froushani, Nasim Hajighahramani, Saied Hosseini-Asl, Farhad Pourfarzi, Rasoul Nemati

**Affiliations:** 1 Department of Microbiology, Faculty of Veterinary Medicine, Urmia University, Urmia, Iran; 2 Digestive Disease Research Center, Ardabil University of Medical Sciences, Ardabil, Iran; 3 Department of Pharmaceutical Biotechnology, School of Pharmacy, Ardabil University of Medical Sciences, Ardabil, Iran; 4 Department of Internal Medicine, Faculty of Medicine, Ardabil University of Medical Sciences, Ardabil, Iran; University of the Witwatersrand, SOUTH AFRICA

## Abstract

**Background:**

The interplay between the gut microbiota axis and host immunity is pivotal in the pathogenesis of inflammatory bowel disease (IBD), an idiopathic inflammatory condition. Molecular mimicry may be at the root of autoimmune and auto-inflammatory diseases, such as IBD, when microbial antigens and host proteins share structural and molecular similarities. However, auto-inflammation can also occur through mechanisms independent of molecular mimicry. The present study focused on the possible involvement of intestinal bacterial heat shock proteins (HSPs) in the immunopathogenesis of IBD as a cutting-edge issue.

**Methods:**

We employed an immuno-informatics approach to evaluate host-microbe interactions and predict the involvement of bacterial HSPs 60, 70, and 90 in IBD via molecular mimicry as our primary objective. The substantial evolutionary conservation of HSPs and their presence in inflammation scenarios propelled our research. To validate our approach, we performed docking and molecular dynamics (MD) simulations on selected HLA-epitope complexes.

**Results:**

Our analysis revealed that all studied bacteria, compared to Homo sapiens, exhibited meaningful sequence similarity and identity of HSPs. Thirteen bacterial species and their corresponding thirteen epitopes derived from HSP counterparts were selected for further investigation. Finally, a specific epitope of human HSP60 and three epitopes of HSP70 demonstrated considerable sequence similarity to their bacterial counterparts, which was further corroborated through MD simulations as a primary outcome. The secondary outcomes encompassed various factors, including assessing residues in the epitope and receptor-binding grooves within the epitope-HLA complex. Based on the secondary data analysis, the co-expression findings suggested that HSP70 could serve as epitopes in eliciting T-cell-mediated autoimmune responses during infections.

**Conclusion:**

The study provided evidence of molecular mimicry between microbial and human HSPs, which could serve as molecular targets for cross-reactive T cells. In addition to considering sequence similarity, our study emphasized the importance of structural interactions as essential factors in cell signaling and immunological pathways.

## 1 Introduction

Spondylarthritis represents a diverse spectrum of diseases characterized by distinct clinical features and varied etiologies, including inflammatory bowel disease (IBD). The immunopathogenesis of IBD involves a complex interaction among genetic, environmental, and microbial factors that leads to the dysregulation of the host immune system. This dysregulation of gut microbiota is associated with an impaired immune response and compromised mucosal barrier integrity, a condition often referred to as barrier disease [1–6]. Recent research has proven the essential role of the gut microbiota in maintaining immune homeostasis and in the development of autoimmune and immune-related conditions [2,3,7,8]. IBD is characterized by chronic autoinflammation, which is frequently linked to dysbiosis, alterations in gut bacterial composition, and a compromised gut barrier contributing to the progression of the disease. The gastrointestinal (GI) environment is a crucial field of research because it represents a connection between nutrition and environmental stimuli related to immune diseases, such as autoimmunity. Hence, understanding the interactions between the host and resident microbes is a key point to consider in elucidating the etiopathogenesis of IBD [2,8–12]. Active research areas include alterations in the microbiota and its metabolic output, the inflammatory cascade triggered by increased intestinal permeability, and identifying specific bacterial species associated with autoimmunity and inflammation [2,9,10,12–15]. The hygiene hypothesis suggests that the cumulative burden of infections throughout an individual's life may contribute to the development of autoimmunity, particularly in those with a genetic predisposition. On the other hand, certain microorganisms may prevent specific autoimmune disorders [4,11,14,16–18]. According to particular research findings on the link between infection and autoimmune disease, the infection triggers an inflammatory reaction to a food-borne antigen, which may lead to autoimmune and auto-inflammatory diseases [9,19].

Individuals with genetic susceptibility may experience autoimmune reactions due to environmental exposure to microorganisms, as well as the similarity between host, pathogen, or commensal microbes, which can compromise self-tolerance. T cell receptor (TCR) degeneration recognizes diverse antigens, facilitating cross-reactivity. The role of the human leukocyte antigen (HLA) complex as a genetic determinant in modulating the immune system is well-established, with particular alleles linked to IBD and autoimmune dysbiosis [1,20–23]. The molecular mimicry hypothesis proposes that similarities in protein sequences between self-proteins and infectious pathogens can lead to cross-reactivity of immune cells, potentially triggering the activation of autoreactive T cells, resulting in the development of an autoimmune response and tissue damage in IBD [2,17,24–27]. However, the exact nature of these interactions remains to be elucidated through clinical and experimental validation. HLAs can help us understand the immunological pathogenesis of illness by presenting epitopes to autoreactive T lymphocytes [1,20,23,27–32]. Among the various proteins implicated in this process, Heat Shock Proteins (HSPs) have emerged as significant due to their evolutionary conservation across species and their role in cellular stress responses, including those preceding IBD onset, such as infection, inflammation, and thermal shock [33–37]. HSPs, particularly HSP60, HSP70, and HSP90, are among the most immunogenic microbial antigens.

The potential amino acid level similarity between bacterial and human HSP raises the hypothesis that molecular mimicry may play a role in the pathogenesis of IBD [17,33,34,38–41]. The detection of Hsp60 and Hsp70 in healthy peripheral blood, along with their phylogenetic conservation, indicates that immune responses to HSP are strictly regulated [40,42,43]. Regulatory activities of immunomodulatory antigen-presenting cells (APCs) may influence the presentation of HSPs to T lymphocytes without additional signals, thereby causing the inactivation of autoreactive T lymphocytes under stressful conditions. HSPs derived from commensal gut bacteria could stimulate autoreactive T lymphocytes, while self-HSP regulatory T cells may develop in the tolerant gut lining [12,33,43]. Therefore, an in-depth study of the HSP and its function and regulation can lead to a promising approach to understanding disease immunopathogenesis and therapeutic discoveries.

The systemic immune disorder also impacts many human organs outside the GI tract, necessitating a cross-disciplinary investigation. The rapidly expanding body of biomedical knowledge has profound implications for clinical practice and patient well-being. Immuno-informatics, a unique interdisciplinary area, is increasingly helping researchers analyze high-dimensional immunological data, providing pathbreaking results for society and medicine. Immuno-informatics enhances our understanding of specific immune responses by studying short immunogenic peptide fragments or motifs, thus overcoming the limitations of traditional experimental research methods. Moreover, the in-silico approach provides a critical foundation for generating hypotheses and designing experiments, ultimately contributing to more effective research outcomes. As a branch of bioinformatics, immuno-informatics plays a considerable role in understanding the pathogenesis of autoimmune and auto-inflammatory conditions and guiding prevention and treatment strategies [26,44]. Current research on IBD focuses on the gut microbiota, their metabolic profile, and the dysregulation of gut-associated lymphoid tissue (GALT) [2,15,45–47]. The pathogenesis of IBD remains an area of ongoing investigation. Recent advancements in computational biology have introduced innovative strategies for vaccine development. Employing immuno-informatics methodologies can accurately predict antigenic epitopes, which may facilitate meaningful practical applications. Adopting immuno-informatics enables considerable reductions in both time and cost barriers while increasing the likelihood of successful immunological outcomes. Integrating immuno-informatics with conventional experimental methods represents a promising strategy for disease prevention and therapeutic outcomes [26,48]. Additionally, exploring the concept of molecular mimicry, a long-standing phenomenon, could yield valuable insights and advance our knowledge in this field, thereby opening new avenues for innovative research. Research has examined the potential of its presence in IBD [2,9,24,25,33,49]; however, this matter has not been thoroughly investigated via detailed immunopathological mechanisms. Our study aimed to expand upon this foundational understanding by exploring the immunoinformatic approach. Our study hypothesizes that the amino acid level similarity between HSPs of certain bacteria implicated in IBD and human HSPs may contribute to the pathogenesis of IBD through molecular mimicry. This hypothesis is grounded in the evolutionary conservation of HSPs and increased expression under stress conditions, such as in the pre-IBD gut environment. In the context of IBD, the concurrent presence of bacterial and human HSPs can lead to the activation of APCs via their Major Histocompatibility Complex class II (MHC-II) molecules. Similarly, GI and intestinal cells could present bacterial HSPs to T cells via MHC class I (MHC-I) pathways, leading to autoreactivity in T cells [12,33,34,43]. The structural investigation into the feasibility of presenting HSPs by MHC-I and MHC-II is noteworthy for understanding which epitopes of HSPs on which HLA molecules may trigger autoreactive T cells. Identifying these epitopes could provide insights into the initiation or acceleration of IBD and may be considered a contributing factor to the immunopathogenesis of the disease. Our study aimed to conduct a predictive immunoinformatic investigation into the molecular-level similarities between host and specific bacteria, including protein and amino acid levels. Additionally, we aimed to predict the particular epitopes that trigger this phenomenon in IBD using an immunological perspective and the dry lab approach. Primarily, our investigation aimed to predict which epitope of human and microbial HSPs could potentially lead to autoreactive T lymphocytes in genetically predisposed individuals. To accomplish this, we utilized immuno-informatics techniques to identify 9-mer and 15-mer epitopes from HSPs of 60, 70, and 90 kDa. This study compares epitopes derived from selected bacterial HSPs to further explore the concept of molecular mimicry as a secondary objective. Through a thorough analysis of sequence similarity and identity at the amino acid level, potential epitopes associated with IBD were identified, establishing a connection between molecular mimicry and the pathogenesis of IBD. Furthermore, we conducted a comprehensive investigation to identify bacterial agents that may play a role in molecular mimicry. This involved examining the similarities between their HSP sequences and those of self-HSPs. We have gained valuable insights that could inform future experimental studies on IBD by characterizing critical epitopes and analyzing their interactions with immune system receptors. This dry lab approach uncovered potential molecular signatures related to IBD pathogenesis and highlighted the importance of immuno-informatics in enhancing our understanding of autoimmune diseases.

## 2 Materials and methods

**Fig 1:**
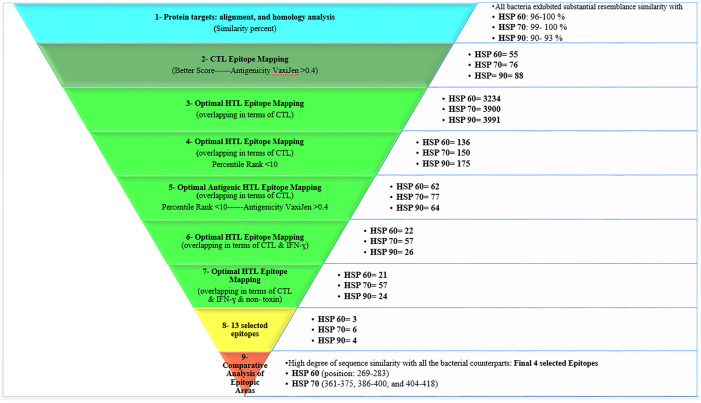
Graphical abstract: Diagram of the workflow of the proposed methodology of the steps used to reach the possible validity of the molecular mimicry hypothesis in IBD disease through human HSPs and bacterial equivalents. Each part of the pyramid shows the steps of the method from top to bottom. The Bullitt shape next to each step of the pyramid provided practical explanations of the study results, as well as the number of epitopes predicted from each of the HSPs presented by HLAs at that step. The workflow of 7 steps of the proposed method to predict the epitopes is as follows: **In step 1**, Protein targets: alignment, and homology analysis (Similarity percent), all bacteria exhibited substantial resemblance similarity with HSP 60: 96-100% (*E. coli* and *S. typhi* revealed 100% similarity); HSP 70: Almost 99- 100% (except *E. coli* with 92% similarity); HSP 90: Almost 90- 93% (except *L. monocytogenes* = 70% and *C. difficile* = 82%. **In step 2**, CTL 9-mer epitopes were selected based on HLA, toxicity, and antigenicity considerations. **In step 3**, the Optimal HTL 15-mer Epitope Mapping (overlapping in terms of CTL) includes HSP 60 = 3234 (presented by MHC-II: related = 1471; not related 1763), HSP 70 = 3900 (presented by MHC-II: related = 1770; not related = 2130), HSP 90 = 3991 (presented by MHC-II: related = 1813; not related = 2178). **In step 3**, the HSP 60-related overlapping HTL epitope with CTL epitope presented by related (to IBD) MHC II HLA includes DRB3*01:01 = 295, DQA1*05:01-DQB1*02:01 = 294, DRAB1*03:01 = 294, DRB1*04:01 = 294, DRB1*13:01 = 294. **In step 3**, the HSP 60-related overlapping HTL epitope with CTL epitope presented by not related (to IBD) MHC II HLA includes: DRB1*01:01 = 295, DRB1*15:01 = 295, DRB1*12:01 = 295, DRB1*13:02 = 295, DRB1*11:01 = 295, DRB1*07:01 = 288. The HSP 70 related overlapping HTL epitope with CTL epitope presented by related (to IBD) MHC II HLA includes: DRB3*01:01 = 355, DQA1*05:01-DQB1*02:01 = 353, DRAB1*03:01 = 355, DRB1*04:01 = 354, DRB1*13:01 = 353. The HSP 70 related overlapping HTL epitope with CTL epitope presented by not related (to IBD) MHC II HLA includes DRB1*01:01 = 355, DRB1*15:01 = 355, DRB1*12:01 = 355, DRB1*13:02 = 355, DRB1*11:01 = 355, DRB1*07:01 = 355. The HSP 90 related overlapping HTL epitope with CTL epitope presented by related (to IBD) MHC II HLA includes: DRB3*01:01 = 363, DQA1*05:01-DQB1*02:01 = 363, DRAB1*03:01 = 363, DRB1*04:01 = 361, DRB1*13:01 = 363. The HSP 90 related overlapping HTL epitope with CTL epitope presented by not related (to IBD) MHC II HLA includes DRB1*01:01 = 363, DRB1*15:01 = 363, DRB1*12:01 = 363, DRB1*13:02 = 363, DRB1*11:01 = 363, DRB1*07:01 = 363. **In step 4**, the Optimal HTL 15-mer Epitope Mapping (overlapping in terms of CTL-Percentile Rank <10) includes HSP 60 = 136 (presented by MHC-II: related = 69; not related = 67), HSP 70 = 150 (presented by MHC-II: related = 90; not related = 60), HSP 90 = 175 (presented by MHC-II: related = 96; not related = 79). **In step 5**, the Optimal HTL 15-mer Epitope Mapping (overlapping in terms of CTL-Percentile Rank <10-Antigenicity VaxiJen > 0.4) includes: HSP 60 = 62 (presented by MHC-II: related = 33; not related = 36), HSP 70 = 77 (presented by MHC-II: related = 65; not related = 33), HSP 90 = 64 (presented by MHC-II: related = 49; not related = 32). **In step 5**, as described in the results section, the candidate 15-mer epitopes were chosen according to study criteria (immunological filters). **In step 8**, 13 epitopes were chosen considering (Table 1) 1- the high dispersion of restricting HLA class II alleles and 2- related percentile rank. **In step 9**, Comparative Analysis of Epitopic Areas, the human HSPs exhibited a high degree of sequence similarity with all their bacterial counterparts, specifically HSP 60 (positions 269-283) and HSP 70 (positions 361-375, 386-400, and 404-418). Note: Several HLAs can present each epitope, so the sum of epitopes of each HSP is not necessarily obtained from the sum of epitopes in complex with related and unrelated HLAs in opposite parentheses. (Receptor, HLA; Ligand, Epitope). Abbreviations: HSP, heat shock protein; MHC, major histocompatibility complex; HLA, human leukocyte antigen; CTL, cytotoxic T lymphocyte; HTL, helper T lymphocyte; IFN-γ, interferon-gamma.

Our primary objective in this study was to investigate the molecular immunopathogenesis of IBD with a focus on exploring potential HSP epitopes. We analyzed antigenic and non-toxic epitopes that could induce Cytotoxic T cell lymphocytes (CTL), Helper T cell lymphocytes (HTL), and Interferon-gamma (IFN-γ). Our investigation involved a comparative analysis of epitopic areas with thirteen bacteria listed in S1 Table. A visual representation of our workflow methodology for epitope calling is manifested in Fig 1. Then, the binding affinity and stability of identified epitopes in the epitope-HLA complex and the exact amino acid residues interacting in the agretope of the epitope and receptor binding pocket of epitope-HLA complexes were analyzed through docking and Molecular dynamics (MD) simulation as part of secondary objectives. In terms of antigen presentation, an agretope refers to a unique area, distinct from the epitope, that engages with the binding groove of the MHC molecule and is recognized by the TCR. This study was approved by the institutional review board (IRB) and the Ethical Committee of Ardabil University of Medical Sciences with IR.ARUMS.REC.1403.295 as ethical code.

**Table 1:** Listing of 13 characteristics of selected epitopes. These include overlapping CTL, HTL, IFN-γ, antigenicity, and non-toxicity. We ranked peptides as strong, mild, and weak based on their binding manner (percentile). The potent binding peptides are represented by a shade of Lt Trellis style pattern cells, the mild ones by a shade of Lt Vertical style pattern cells, and the weak ones by white cells. The epitope thresholds for classification as strong, mild, and weak are less than 2% for strong, between 2% and 5% for mild, and between 5% and 10% for weak. The amino acids underlined in the “HTL Epitope Sequence” column represent the overlapped core amino acids with the CTL epitope. The machine learning prediction algorithm of the studied server classifies positive numbers in the SVM score as IFN-γ cytokine epitope (seventh column of the table) and negative numbers as non-toxins (eighth column). The abbreviations used in the table are HSP (heat shock protein), HLA (human leukocyte antigen), CTL (cytotoxic T lymphocyte), HTL (helper T lymphocyte), IFN-γ (interferon-gamma), SVM (Support vector machine).

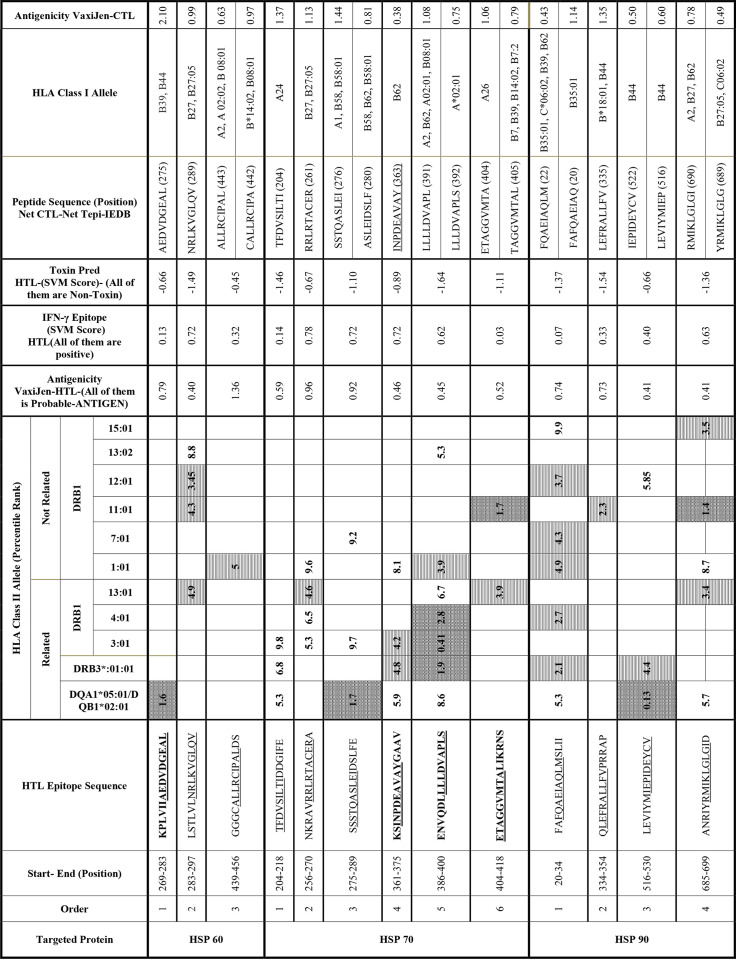

### 2.1 Physicochemical, 2D structure, alignment, homology, and phylogenetic analysis of protein targets

Comparing the amino acid sequences of HSPs of Homo sapiens and bacteria using bioinformatics methods can clarify the degree of molecular mimicry between these HSPs and thus help us to understand the immunopathogenesis of IBD. In this context, the sequences of HSPs 60 (GroEL), 70 (DnaK), and 90 kDa (htpG) were acquired from the National Center for Biotechnology Information (NCBI) database. These sequences are presented in FASTA format and represent the peptides of Homo sapiens and pathogenic microorganisms at the amino acid level, serving as sources of both self- and non-self-peptides (S1 Table). For further details on NCBI, visiting the website at http://www.ncbi.nlm.nih.gov/ was recommended. The studies indicated the potential role of all the investigated bacteria via their HSPs or other proteins and mechanisms in IBD immunopathogenesis and implicated them in common GI infections or other systems [4,5,9–11,15,33,35,41,46,47,50–56]. These bacteria mentioned in the literature review concerning IBD were listed in S1 Table. We utilized the Basic Local Alignment Search Tool (BLAST) to align the collected sequences. For more information about BLAST, please visit http://blast.ncbi.nih.gov/. In our research effort, we utilized the online service T-Coffee of the Centre for Genomic Regulation (CRG) in Barcelona to analyze the structural features of expresso-alignments. Using this sophisticated tool, we efficiently aligned protein sequences and accurately determined the percentage of HSP homology in the species studied [57]. The NCBI FASTA format of the sequences was then aligned with various sequences using the Clustal Omega tool in UniProt to determine target protein conservation without adjusting any parameters.

Hits on the target proteins in the human proteome were also investigated. For this purpose, we utilized the BLASTP (protein-protein BLAST) server in conjunction with the Reference Proteins (RefSeq protein) database, as described in “Compositionally Adjusted Substitution Matrices.” The value of E, the expected threshold, was maintained at 0.05 [58]. Additionally, multiple sequence alignments were performed using CLUSTAL O (1.2.4) from UniProt for phylogenetic analysis, which examined their evolutionary divergence. (www.uniprot.org/align/clustalo).

The ExPASy ProtParam tool was used to examine the physicochemical characteristics of the target proteins (http://web.expasy.org/protparam/). Antigenicity was evaluated using VaxiJen 2.0, with an antigen prediction cutoff of 0.4 (http://www.ddg-pharmfac.net/vaxijen/VaxiJen/VaxiJen.html) [59,60].

Currently, DeepTMHMM, as a Deep Learning Model, is the most comprehensive and efficient method for predicting the topology of alpha-helical and beta-transmembrane proteins using deep neural networks. The scientific community greatly benefits from the ability of DeepTMHMM to scan whole proteomes for both kinds of transmembrane proteins. In this study, the DeepTMHMM 2.0 server was used for the cellular topology of each HSP (https://dtu.biolib.com/DeepTMHMM) [61].

Conserved domains as protein families were classified through InterPro 92.0, a part of the ELIXIR infrastructure (https://www.ebi.ac.uk/interpro/search/sequence/) [62]. The physicochemical characteristics of the target proteins were analyzed to describe the anticipated epitopes by utilizing the ExPASy ProtParam tool (Available at http://web.expasy.org/protparam/).

We used Position-specific Iterated BLAST (PSI–BLAST) to identify homology between distant species and confirmed the results with BLASTP. The access link is (https://blast.ncbi.nlm.nih.gov/Blast.cgi) [63,64]. We used the accession number from S1 Table to perform PSI–BLAST with different initial settings compared to BLASTP. We chose the Reference Proteins database (Refseq protein). We selected the settings for the bacteria mentioned in the Organism section to ensure a targeted search and exclude irrelevant species, such as plants and animals. We used the BLOSUM 45 algorithm for our study, as it’s better to set a lower value than the default 62 when comparing sequences from distant species.

### 2.2 Structural similarities of HSPs and their counterparts

In the first step, retrieval and analysis of desired human and bacterial proteins were performed using their UniProt ID in the UniProt database. All human proteins have experimentally determined structures, obtained primarily by cryo-EM, that effectively cover their sequences. Before starting the structure alignment, a comprehensive quality control of the structures was carried out to confirm their high quality. Special attention was paid to the careful testing of Ramachandran’s cases and the minimization of the energy resulting from the AlphaFold. It was ascertained that all the obtained PDB files met these criteria, as no outliers were revealed in the Ramachandran plot during the Procheck analysis, and the structures were found to be acceptable in terms of errat. Only HSP60 and HSP70 from Mycobacterium tuberculosis (strain ATCC 25618/H37Rv) had experimental structures among the microbial proteins. As no experimental structure for bacterial HSP90 was available, the AlphaFold structure [65,66] for HSP90 from Campylobacter jejuni (strain RM1221), with the closest sequence similarity, was utilized.

In the Chimera software, the respective human and bacterial PDB files were initially opened [67]. Subsequently, the MatchMaker tool was utilized with the default settings: the Needleman-Wunsch algorithm, the BLOSUM-62 matrix, and a matching threshold of 2 Å to align all chains between the proteins. The software automatically identified the best-matching chain pair for files containing all chains of the target protein. Irrelevant chains were removed from files that contain a specific target protein chain. The RMSD scores between pruned atom pairs (MatchMaker) for the most similar chains from both proteins were calculated. This process provided the RMSD scores between the matched chains, which were then used to identify and retain similar chains for further analysis. Sequence identity, comparable to BLAST results, was computed. The Match - > Align tool was then employed with default settings and circular permutation enabled to align these similar chains and produce the Overall RMSD (a more robust metric than the previous RMSD), Structural Distance Measure (SDM) [68], and Q-score [69]. SDM is a linear measure of structural dissimilarity, whereas the Q-score indicates the level of agreement between different superpositions. The SDM cutoff does not indicate homology but refers to the computational algorithm. Specifically, the SDM method uses a cutoff threshold of five to sample and measure atomic positional differences. As stated on the MatchAlign help page:

(https://www.cgl.ucsf.edu/chimera/docs/ContributedSoftware/matchalign/matchalign.html). These metrics are pivotal for normalizing RMSD and assessing structural similarity, which is influenced by the cutoff distance used for residue equivalence, as well as the quality of superposition. This provides valuable insights into the structural characteristics of the aligned proteins. Also, the TM-score from RCSB was used for further structural alignment analysis [70].

### 2.3 Epitope mapping or calling

The analysis of epitope calling involved examining potential HLA-restricted epitopes using the Immune Epitope Database and Analysis Resource (IEDB), available at http://www.iedb.org [71,72]. Our investigation led us to select epitopes with the highest binding scores and lowest IC50 for further examination. We found that the identified epitopes had a preferred affinity for specific MHC-I and MHC-II molecules. These immunodominant peptides were compared to the HSP 60, 70, and 90 kDa sequences of particular bacteria mentioned in S1 Table.

#### 2.3.1 CTL epitopes.

We used three servers for 9-mer CTL epitope identification, including NetCTL.1.2, IEDB, and NetTepi 1.0. We used the NetCTL 1.2 server to predict the 9-mer epitopes restricted by human MHC-I supertypes A1, A2, A3, A24, A26, B7, B8, B27, B39, B44, B58, and B62 [73]. The criteria for identifying epitopes were based on “transporter associated with antigen processing” (TAP) transport efficiency and proteasomal C-terminal cleavage, with cutoffs of 0.75, 0.05, and 0.15 for each. Additionally, we identified the restricted epitopes by HLA Class I alleles A*02:01, A*03:01, A*58:01, B*35:01, B*51:01, B*58:01, B*27:05, B*08:01, B*14:02, B*35:03, B*18:01, C*06:02, and C*14:02 using IEDB, which employed the consensus 2.18 prediction method. A low consensus score indicated a good binder [71,72]. The NetTepi 1.0 server, developed by DTU Health Tech, was used to identify epitope restricted by HLA Class I alleles A*02:01, B*35:01, B*58:01, B*27:05, and A*03:01, based on affinity-based scoring of T lymphocyte stability. We assigned a default relative weight of 0.16 to the stability prediction and 0.10 to the propensity prediction of T cells. The following link is provided: https://services.healthtech.dtu.dk/service.php?NetTepi-1.0 [74,75]. The HLA Class I epitopes identified in this study encompass 90 percent of the world’s population [76]. We selected peptides with a consensus score of equal to or less than 2 for further investigation, considering them favorite binders with a cutoff of less than 2. The CTL 9-mer epitopes, sequences, and numbers were retrieved from the integrated and reviewed results of three databases.

#### 2.3.2 HTL epitopes.

The 15-mer epitopes were restricted by different alleles of HLA Class II DRB1, including 01:01, 07:01, 11:01, 12:01, 13:02, and 15:01 were found to be unrelated to IBD. However, they encompassed almost 95 percent of the population and established a connection between HLA and IBD. The IEDB consensus technique was employed to estimate the following alleles: DRB1*03:01, 04:01, and 13:01, DRB3*01:01, and DQA1 05:01/DQB1 02:01 [77,78]. The examined HLA Class II epitopes cover 95 percent of the global population [76]. Peptides were classified into three percentiles: strong, mild, and non-binding, according to their strength. The thresholds used for classification were 2%, 10%, and greater than 10%, respectively [79].

#### 2.3.3 Overlapping, antigenicity, and toxicity of T-cell epitopes.

Epitopes restricted by multiple HLA alleles can play a significant role in the pathogenesis of IBD. These epitopes could trigger a strong immune response in the host. This study identified epitopes that exhibit high binding affinity to multiple HLA molecules. The presence of essential and integral sequences in overlapping epitopes allowed for the activation of CTL and HTL cells [79]. To conduct a more thorough examination, this study focused on 15-mer HTL epitopes with a high binding affinity that overlapped with analogous or comparable CTL epitopes, using the core 9-mer sequence as a basis, and combined them into a single unified peptide fragment. We then used VaxiJen v2.0 (adjusting for the bacteria) to determine the antigenicity of the predicted epitopes, selecting only those with scores below 0.4 as the threshold [59,60]. To scan for toxic motifs in proteins, we utilized the ToxinPred 2.0 prediction module (https://webs.iiitd.edu.in/raghava/toxinpred/protein.phpp) [80,81] and analyzed based on the previous research [79].

#### 2.3.4 Prediction of IFN-γ-inducing epitopes.

IFN-γ, a characteristic cytokine of both the innate and adaptive immune systems, can induce immune regulatory, anti-tumor, and antiviral actions. Identifying IFN-γ-inducing epitopes is fundamental for understanding the potential pathogenesis of IBD [9,82,83]. The online prediction server, IFNepitope, predicted epitopes associated with IFN-γ in target proteins that bind to MHC-II molecules (https://webs.iiitd.edu.in/raghava/ifnepitope/predict.php). In this investigation, the “Motif and SVM hybrid” technique and the “IFN-gamma versus non-IFN-gamma” prediction model were chosen to predict IFN-γ epitopes [83]. The only HTL/MHC-II allele–HSP epitope complexes (IFNepitope usage for just MHC-II binders) associated with IBD that exhibited positive outcomes through this server were selected for subsequent assessment.

#### 2.3.5 Final selected epitopes.

We selected epitopes from previous steps based on their HLA compatibility, toxicity (evaluated by the ToxinPred server), and antigenicity (assessed by the VaxiJen v2.0 server for bacteria). In step 8 of Fig 1, our approach to identifying epitopes restricted by most HLAs examined was systematic and data-driven. We provided a painted epitopic area and detailed information on this systematic process ins S1 File (Excel file), with each sheet corresponding to one of the HSPs under investigation. We listed all HLAs tested in the first row, while the adjacent columns contained the epitopes represented by their amino acid sequence numbers. To facilitate visual analysis and rapid identification of broadly reactive epitopes with HLA, we implemented a color-coding scheme based on the affinity of each epitope for binding to its respective HLA molecule. Finally, regions with high color density, indicating binding to multiple HLA types, were highlighted as epitopic areas for further analysis.

In addition, the first cell of each highlighted row, which represented a 15-mer epitope for HTL, contained the percentile rank of the predicted epitope binding affinity with HTL. A number ranging from zero to ten was assigned, with lower values indicating a stronger predicted binding affinity. The selection of epitopes for subsequent analysis was based on the color density and the best (lowest) percentile rank score for binding affinity. This dual criterion prioritized epitopes with the broadest HLA restriction profile and the highest binding affinity. In this epitope mapping step, we aimed to select epitopes that are most likely to be present in a diverse human population, thereby improving the relevance and applicability of our findings to IBD pathogenesis.

### 2.4 Comparative analysis of epitopic areas

A BLASTP server was used to identify high sequence homology between self-HSPs and HSPs of thirteen bacteria. Based on the results, thirteen HLA-restricted human HSP epitopes were selected [58]. This step was conducted based on previous studies [36,37].

### 2.5 Virtual screening

#### 2.5.1 Analyzing the HLA-epitope complex interactions by computational modeling and docking.

To generate three-dimensional (3D) structures and PDB files of the epitopes, we utilized the PEPFOLD3 server. This server predicts the conformational structures of peptides based on their amino acid sequences, as outlined in the sources [84,85] (https://bioserv.rpbs.univ-paris-diderot.fr/services/PEP-FOLD3/). We obtained X-ray crystallographic structures of the common HLA alleles in humans from the Protein Data Bank (PDB). We eliminated previously bound ligands and minimized their energy. The HLA class I alleles and their corresponding PDB IDs in parentheses were as follows: HLA-B*44:02 (3KPM) [86], HLA-B*15:01 (B62/ 5TXS) [87], HLA-A*02:01 (6TDS) [88], and HLA-B*14:02 (3BXN) [89]. Additionally, we utilized HLA class II alleles, including HLA-DRB1*04:01 (4MD4) [90], HLA-DQ2 (DQ B1*02) 6U3M [91], DRB1*01:01 (7YX9) [92], and DRB1*11:01 (6CPL) [93]. All selected HLA alleles were identified as originating from the Homo sapiens organism, with no observed mutations, and were analyzed using the X-ray Diffraction method at a resolution of less than 2 angstroms (Å), displaying zero Ramachandran outliers (except for 6CPL, which had a 2.45 Å resolution).

To better understand the patterns of protein-protein interactions, molecular docking analysis was conducted using the LZerD (Local 3D Zernike descriptor-based protein Docking) Web Server (https://lzerd.kiharalab.org/upload/) to examine HLA allele interactions with screened epitopes. A refined model was determined to elucidate the connection between epitopes and HLA molecules within the peptide binding groove. The specific details of this model may vary according to cluster size [94]. PyMOL version 2.0, a molecular graphics system developed by Schrödinger, was used to assess the interaction patterns of the docked complexes. To identify the active sites of HLAs’ 3-dimensional protein structures, we employed DeepSite, a protein-binding site predictor that utilizes 3D-convolutional neural networks to evaluate protein (https://www.playmolecule.com/deepsite/) [95]. We also utilized the PDBsum online service (https://www.ebi.ac.uk/pdbsum) provided by the European Bioinformatics Institute (EMBL-EBI) in Cambridge. Specifically, we used the Ramachandran plot statistics feature to analyze the docking structures of 4 HTL and 4 CTL complexes. EMBL-EBI is recognized as a renowned collaborating center for the management and analysis of large-scale biological data [96].

#### 2.5.2 MD simulation analysis.

The CHARMM-GUI server was used to create the simulation system [97–99]. In the first step, the protein-ligand complex structures resulting from the docking process were generated in PDB format using PyMOL software. Subsequently, all of these structures were posted to the CHARMM-GUI website. The primary structure of the protein and the ligand were chosen, and any missing atoms and areas were modeled using an automated process. The dimensions of the solution system were selected to be rectangular, ensuring that the forces exerted on the particles closely resemble those of the fluid mass in real-world settings.

A standard value of 10 Å was employed to represent the separation distance between the boundaries of the biomolecule and the boundaries of the water box. Potassium chloride (KCl) salt ions, possessing a concentration of 0.15 M, were introduced into the system to achieve electrical neutralization, which closely approximates the physiological ion concentration. Cl- and K+ ions were quantified using the ion-accessible volume (V) and total system charge (Qsys). We employed the Particle-Mesh-Ewald method (PME) to effectively counteract the effects of long-range electrostatic interactions [100]. The Charmm36 force field was implemented to construct the topology and parameter files [101]. Finally, all generated components were successfully merged, and the system was thoroughly tested to ensure its structural soundness. The generated files were applied as input data to calibrate and run the simulation. The energy minimization process was performed using the PEM decreasing slope approach, and it was continued until the maximum force reached a value less than 1000 KJ/mol/nm. Achieving balance was frequently accomplished using a two-step approach [100].

We began with an initial phase involving a system of constant particle numbers, volume, and temperature (NVT). The duration of this phase varied depending on the system’s composition. In the NVT ensemble, the temperature was stabilized until it reached a steady state (plateau) at the target value. If the temperature was unstable, we extended the time. Generally, we found that 50–100 picoseconds (ps) were sufficient. Once the temperature was stabilized, the experiment proceeded to the second equilibration stage, and the system pressure and, thus, density were stabilized using an NPT apparatus. This means the number of particles, pressure, and temperature remain constant before data collection. A Parrinello-Rahman barostat was used to add pressure [102]. Finally, we simulated 100 nanoseconds using the CHARMM36 force field to solve the equations of motion for the system, which contained the protein and ligand [101].

The simulation stability and energy balance were assessed by analyzing temperature, pressure, potential energy, and total energy following convergence. The flexibility and dynamism of the simulated HLA-Epitope complexes were revealed by studying their radius of gyration (Rg). Measuring the molecule’s shape was possible by tracking the radius of gyration at any given time. The Rg was then compared to the hydrodynamic radius that could be achieved and calculated using gmx gyrate. The Rg diagram was a valuable tool for better understanding the molecule’s overall expansion, the compactness index of the protein structure, and the protein’s stability. It was necessary to ensure that the simulation reached an equilibrium state before proceeding with further analysis.

**2.5.2.1 RMSD analysis of HLA-epitope complexes from the initial structure during simulation**

In bioinformatics, the root-mean-square deviation (RMSD) is a valuable metric for determining the average distance between atoms of superimposed proteins, with a focus on the backbone atoms. After careful analysis of energy parameters and validation of simulation accuracy, RMSD plots were generated for the selected HLA-epitope complexes and epitopes. These diagrams confirmed the thermodynamic stability of the system through the plateau region, representing thermodynamic equilibrium. Therefore, these plots were considered a reliable tool for determining the appropriate time interval for further analysis. The RMSD analysis provided insight into the stability and flexibility of the protein during the simulation by comparing the state at the beginning (time zero) to the state at the end of the 100 ns simulation.

**2.5.2.2 RMSF analysis of HLA-epitope complexes during the simulation**

Through root-mean-square fluctuation (RMSF) analysis, protein flexibility was analyzed by measuring fluctuations in the average position of individual atoms or residues. This analysis enabled us to identify protein regions with higher RMSF values, which show greater flexibility during the simulation. We utilized the gmx rmsf tool of the Gromacs program to calculate the RMSF and generate a graph showing changes concerning the number of residues. This structural analysis allowed us to pinpoint the amino acids that contribute the most to the movement of the molecule and identify the most flexible parts of the protein structure [103]. Our analysis included calculating RMSF values for all HLA residues and epitopes in the complexes.

**2.5.2.3 Analysis of hydrogen bonds**

In MD simulations, it was necessary to determine the number of hydrogen bonds between the protein and its attached ligand to comprehend the complexity of the protein-ligand complex. The presence of a hydrogen bond was determined by measuring the distance and angle between an acceptor and a hydrogen donor. Our analysis focused on the impact of hydrogen interaction on the structure and stability of the HLA-Epitope complex, utilizing a gmx hbond and a 3.5 Å distance cutoff for H-bond analysis.

**2.5.2.4 Analyzing hydrophobic interactions**

Hydrophobic interactions played a considerable role in stabilizing the HLA-Epitope complex. The LigPlot+ program can be used to verify this. To confirm the presence of these interactions, the final frame of the simulation path for each complex was analyzed [104].

### 2.6 Random forest and analytical strategy for evaluating the host and meta-expression patterns

In this study, to investigate the co-expression of chaperone and HSP genes in the host’s Gut and gut microbiome, we applied secondary data analysis through “The Inflammatory Bowel Disease Multi-Omics Database” (IBDMD). The following link is provided: https://ibdmdb.org/. We utilized 146 samples from the human gut transcriptome data, comprising 95 samples from individuals with IBD and 51 samples from non-IBD individuals. Additionally, we analyzed 78 samples for the metatranscriptome, including 49 IBD and 29 non-IBD samples (S2 File, Sheets 2, 3, and 4). All samples were obtained from the IBDMDB database. We filtered the samples to include only those from individuals aged 18 and above, as IBD affects individuals differently based on age (i.e., individuals aged 18 and above). Information from various literature suggests that IBD manifests differently in individuals under the age of 18 compared to those over 18 [105,106]. The host transcriptome data was a read count table, representing the number of reads mapped to human genes. After filtering and normalization using the DESeq2 package, we ended up with 47034 genes [107]. Furthermore, we analyzed 78 paired-end metatranscriptome samples using Bowtie2 software [108] and mapped them to the integrated gene catalog (IGC) gene catalog [109]. After processing the data using the DESeq2 package, we identified 2576791 microbial genes. During processing and raw data extraction, as the read count was conducted through mapping using Bowtie2, we removed the genes mapped to fewer than 10 reads. Then, normalization was performed using DESeq2. The method used in this section is derived from this study [110].

After identifying the classification features of IBD and non-IBD samples, a challenge was created due to the large number of these features. Overall, the dataset comprised 146 host transcriptome samples, containing 47034 features, and 78 metatranscriptome samples, with 2576791 features, indicating the breadth of the dataset. To address this, we utilized the Python 3.12 pyswarms package for feature selection using particle swarm optimization [111]. Subsequently, we obtained 4,283 features for the host transcriptome sample and 22,741 features for the metatranscriptome (S2 File, Sheet 1). We deployed the Random Forest (RF) algorithm as a machine learning model in the Scikit-Learn Python 3.12 package for sample classification and division into training and test sets at an 80:20 ratio. We then executed the RF algorithm classifier on the data and reported the best evaluation results detailed in S5 Table. The results showed that the RF algorithm classified the metatranscriptome and host transcript samples with 93% and 90% accuracy, respectively, which is an acceptable accuracy. Other evaluation parameters, including F1-score, also showed satisfactory results.

After analyzing the host transcriptome samples and referring to existing literature, we identified two gene groups: HSPs, comprising 82 genes, and the highly correlated IBD group, consisting of 111 genes (S3 File). The databases used for high-correlated genes with the IBD analysis included the Gene and Autoimmune Disease Association Database (GAAD) [112], DisGeNET databases [113] (available at Disgennet.org), SNPedia.com [114], Ensembl.org [115], Omim.org [116], and Varsome.com [117]. Of the 4283 genes identified through the feature selection algorithm, nine were common in the HSP and IBD groups. At the same time, 23 were common in the high-correlated group with the IBD group (S2 File, Sheet 6). To assess the expression correlation of these genes with a specific number of genes identified in metatranscriptome samples, we required individuals who had both host transcriptome and metatranscriptome samples available. Fortunately, we identified 13 such individuals in the IBDMDB database (S2 File, Sheet 5). To comprehensively evaluate the relationship between human and bacterial HSP expression levels and co-expression patterns, a Pearson correlation analysis was conducted using the NumPy package in Python 3.12 [118,119].

In the feature selection process, we identified nine types of HSP genes, with only HSPA6 and HSPA13 belonging to the HSP70 family and HSP90B1 as a member of the HSP90 protein family. Subsequently, we selected host transcriptomes from the IBDMDB based on the presence of acceptable transcripts, non-pseudogene status, and relevance to our study’s proteins (HSP60, 70, and 90). These genes were then analyzed for their correlation using a Pearson correlation coefficient of 0.8 or greater, as detailed in S4 File Sheet 3.

Moving forward, the IGC database (db.cngb.org) (dataset available at https://db.cngb.org/microbiome/genecatalog/genecatalog_human/) was used, containing approximately 9.8 million gut microbiome genes, for biological annotation [109]. The following link is provided: https://ftp.cngb.org/pub/SciRAID/Microbiome/humanGut_9.9M/GeneAnnotation/IGC.annotation_OF.summary.gz. Each transcriptome was assigned to a specific Kyoto Encyclopedia of Genes and Genomes (KEGG) orthology (KO) group, and we investigated their metabolic pathways and related KEGG Enzyme Commission (EC) numbers [120]. Subsequently, we obtained reactions associated with each EC number in the KEGG database based on the studies [110,121]. Notably, we identified bacterial transcripts with biological connections to folding, chaperone, and stress mechanisms, and these were highlighted in red font for emphasis.

## 3 Results

### 3.1 Homology analysis, sequence alignment of targets

We examined the proteomes of microorganisms related to IBD in the literature review for protein sequences with identities (exact match of amino acids) and similarities (conservative substitutions) exceeding 35% and 82%, respectively, using the BLASTP tool from NCBI and T-Coffee Express. These numbers represent the minimum percentage of similarity for the bacteria studied. Therefore, the lowest rate was reported, and all bacteria were selected [48,122]. We summarized the results related to the identities and similarities of human HSPs 60, 70, and 90 with the equivalent bacterial protein in S1 Table. S2 Table shows the results of aligning the sequences of human HSPs with those of HSPs from different bacteria at the amino acid level. S1 Table presents the findings, accession numbers, and protein ID of all the target proteins in the current investigation. Our analysis revealed that the amino acid sequences of microbial HSPs and HSPs in the human proteome were comparable to a great extent, suggesting that human HSP60, 70, and 90 are involved in antigen recruitment. These comparable epitopes were potentially recognized as a result of an immune response aimed against microbial antigens. We employed a comprehensive comparison strategy to identify which microbes were most likely to act as molecular mimics and induce or relapse IBD. Our findings in S1 Table show that all the bacteria exhibited substantial resemblance similarity with HSP 60 (96–100 percent), HSP 70 (99–100 percent, except *Escherichia coli O157:H7* with 92 percent similarity), and HSP 90 (90–93 except *Listeria monocytogene*s and *Clostridium difficile* with 70 and 82 percent similarity respectively). *E. coli O157:H7* and *Salmonella typhi* showed 100% similarity with HSP 60.

According to the data presented in S3 Table, the complete amino acid sequence of HSP 70 was identified as the most antigenic peptide on the VaxiJen 2.0 server, closely followed by HSP 90 and HSP 60, with antigen values of 0.5168, 0.5009, and 0.4997, respectively. All of them (three) were probable antigens with a threshold of 0.4. Other physicochemical variables were evaluated and summarized in S3 Table, including molecular weight, theoretical pI, secondary structure, and half-life in human reticulocytes. All studied HSPs were structured as globular proteins and were located within the cell. Through InterPro analysis, it was determined that the conserved amino acid regions of HSP60 were 430–441; for HSP70, they were 9–16, 197–210, and 334–348, and for HSP90, they were 38–47. Additionally, a phylogenetic analysis was conducted using CLUSTAL O (1.2.4) to align multiple sequences from UniProt (S1 Fig) and calculate their evolutionary distance. Interestingly, a closer common ancestor between Homo sapiens and L. monocytogenes than other bacteria were observed in the case of HSP 90. Notably, the conservation of protein sequence during evolution was observed between HSP60 and HSP70 of E. coli and Shigella dysenteriae, as well as in HSP90 of *E. coli* and Streptococcus pneumoniae. Finally, S2 Fig characterizes the topology analyzed through DeepTMHMM.

The results of PSI–BLAST and its iterations are presented in S5 File. In the third and fifth rounds of iteration, respectively, HSP60 and HSP70 showed stable results. HSP90 did not yield any new results in subsequent iterations. Each HSP and iteration result is shown separately on a different Excel worksheet. The BLAST tree or Distance tree, which shows the homology and distance of species based on the mentioned protein, is also included. The specificity of BLASTP surpasses that of PSI–BLAST. When BLASTP fails to demonstrate homology, utilizing PSI–BLAST becomes imperative to prevent the exclusion of distant homologs. However, in this particular investigation, BLASTP yielded satisfactory homology. Despite the unnecessary nature of PSI–BLAST, it was executed to corroborate the findings. The results of PSI–BLAST essentially confirmed those of BLASTP (S1 Table).

### 3.2 Structural similarities of HSPs and their counterparts

**Fig 2:**
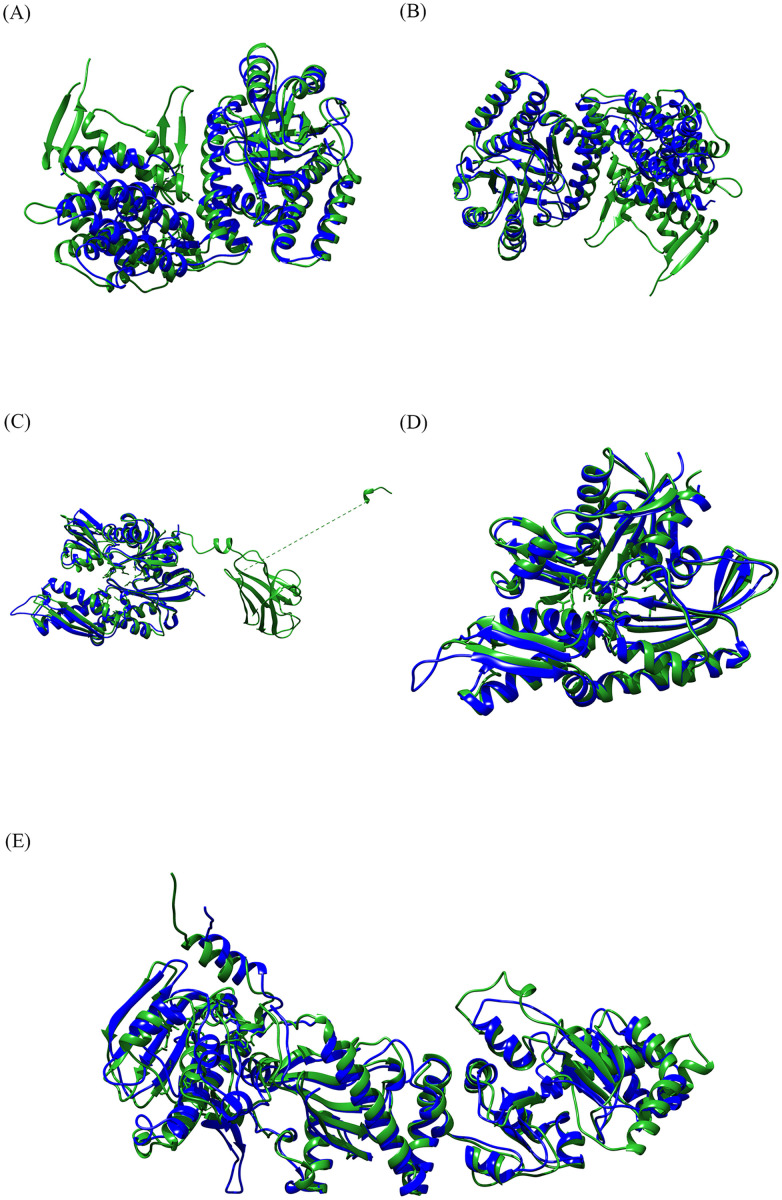
3D structural alignment visualization of HSPs and their counterparts using Chimera. Human protein chains are represented in green, while bacterial proteins are represented in blue. The figure illustrates a clear correspondence between the alpha-helix and beta-sheet structures of human HSPs and their bacterial counterparts. The structural alignment presented indicates a noteworthy similarity between each pair: (A) HSP60 (8g7lH) aligned with GroEL as the HSP60 counterpart of *M. tuberculosis* (3rtkA). (B) HSP60 (8g7lJ) aligned with GroEL as the HSP60 counterpart of *M. tuberculosis* (3rtkB). (C) HSP70 (7kw7C) aligned with DnaK as the HSP70 counterpart of *M. tuberculosis* (6w6eI). (D) HSP70 (7kw7D) aligned with DnaK as the HSP70 counterpart of *M. tuberculosis* (6w6eI). (E) HSP90 (7zubA) aligned with htpG as the HSP90 counterpart of *C. jejuni* (AF-Q5HVP5-F1-model_v4). The PDB ID is provided, with the final uppercase letter indicating the respective chain written in parentheses. The RMSD results from the MatchAlign tool in Chimera for pairwise structural alignment show structural similarity, especially for HSP70 pairs. Abbreviations: Three-dimensional (3D); HSP (heat shock protein); *Mycobacterium tuberculosis* (*M. tuberculosis*); *Campylobacter jejuni* (*C. jejuni*).

The best matches were selected based on S6 File and Fig 2, as previously described in the alignment of multimeric files. RMSD values less than one indicate a definite homologous relationship between the two HSP70-related chains, indicating higher structural similarity. Additionally, RMSD values less than 2 suggested high similarity, as observed for the next two proteins, HSP60 and HSP90. The RCSB alignment RMSD served as the homologous criterion for the TM score algorithm. All target structures exhibit acceptable homology, as values greater than 0.2 indicate significant similarity, while those above 0.5 suggest high homology. Additionally, the acceptable score for both structural and sequence alignment was achieved using the MatchAlign sequence alignment score. Significantly, although the scores for structural alignment were approximate and probabilistic, background knowledge of two proteins with similar functions and a known homologous relationship can raise the score, indicating their homology.

### 3.3 Overlapping epitope mapping selection

In Fig 1, we identified 55, 76, and 88 CTL antigenic epitopes for HSP 60, 70, and 90, respectively. To identify potential immunodominant HTL epitopes, we used the IEDB consensus method and selected the highest-scoring epitopes restricted by HLA alleles. Additionally, we identified the epitopes and overlapping epitopes based on the criteria of our study. After applying immunological filters to the predicted epitopes, we selected those with favorable binding affinities and filtered them to isolate the most effective ones. The optimal epitopes met our criteria of being conserved, immunogenic (in terms of antigenicity), non-toxic, and overlapping in their recognition by CTL, HTL, and IFN-γ. They also had high scores, percentile ranks, affinities, and compatibility with restricting HLA alleles. S7-S10 Files contain the list of the candidate epitopes.

For HSP 60, 70, and 90, we identified 62, 77, and 64 overlapping antigenic HTL epitopes, respectively (overlapping in terms of CTL). However, the overlapping epitopes concerning IFN-γ and non-toxic reduced these numbers to 21, 57, and 24, respectively. We selected 13 epitopes based on their high dispersion of restricting HLA class II alleles and related percentile rank (Fig 1 and Table 1).

### 3.4 Final epitope selection through comparative analysis of epitopic areas

This study compared the structure and binding properties of bacterial and human HSP epitopes using BLASTP to identify potential matches and similarities. The findings presented in S11 File indicate that the sequences of human HSPs of 60 and 70 kDa, as well as their bacterial counterparts, are highly similar. Notably, all analyzed bacteria showed similar similarity patterns. There was meaningful similarity observed for HSP 60, 70, and 90 in the restricted epitopes by DQA1*05:01-DQB1*02:01. No lack of homology was observed among any of the studied HLAs populations for any of the studied HSPs, suggesting that the DQA1–0501-DQB1–0201 alleles were the most restrictive for the majority of the putative immunodominant peptides that resemble the epitopes found on human HSP60.

The sequence of human HSP 70 at positions 204–218 exhibited a high level of similarity with most bacterial counterparts, except for two – *Campylobacter jejuni* and *Helicobacter pylori* – that did not show expected similarity. Human HSP 90, on the other hand, displayed a punctual degree of similarity with most bacteria in certain positions (20–34 and 334–354) but lacked homology with four selected epitopes of *C. difficile* and *L. monocytogenes*. No significant similarity was found between the studied bacteria and the identified epitopes from the previous step, including the second (256–270) and third (275–289) selected epitopes of HSP 70 kDa, as well as the third (516–530) and fourth (685–699) selected epitopes of HSP 90 kDa. (Numbering according to the amino acid position).

One of the most remarkable findings was the meaningful sequence similarity between human HSP60 and 70 and their bacterial counterparts, with an extreme resemblance in the HSP60 (269–283) and HSP70 (361–375, 386–400, and 404–418) regions. The HLA DQA1*05:01-DQB1*02:01 allele restricted the final selected epitope of HSP 60, while the HLA-DRB1 alleles restricted three areas of HSP 70. Table 2 shows these similarities with selected epitopes.

**Table 2:** The level of similarity between sequences of human HSPs at certain positions, as selected epitopes, and their bacterial counterparts. The abbreviations used in the table are HSP (heat shock protein), *C. jejuni* (*Campylobacter jejuni*), *C. difficile* (*Clostridium difficile*), *E. coli* (*Escherichia coli*), *H. pylori* (*Helicobacter pylori*), *K. oxytoca* (*Klebsiella oxytoca*), *L. monocytogenes* (*Listeria monocytogenes*), MAP (*Mycobacterium avium paratuberculosis*), *M. leprae* (*Mycobacterium leprae*), *M. tuberculosis* (*Mycobacterium tuberculosis*), *S. typhi* (*Salmonella typhi*), *S. dysenteriae* (*Shigella dysenteriae*), *S. pneumoniae* (*Streptococcus pneumoniae*), *Y. enterocolitica* (*Yersinia enterocolitica*).

HSPs	Selected Epitopes (positions)	High similarity	Lack Similarity
HSP-60	First 269–283	All of them	
	Second (283–297)	*C. difficile, L. monocytogenes*	11 bacteria from 13 ones
	Third (439–456)	MAP, *M. leprae*	11 bacteria from 13 ones
HSP-70	First (204–218)	11 bacteria from 13 ones	*C. jejuni*, *H. pylori*
	Second (256–270)	None of them	
	Third (275–289)	None of them	
	Fourth (361–375)	All of them	
	Fifth (386–400)	All of them	
	Sixth (404–418)	All of them	
HSP-90	First (20–34)	*C. jejuni*, *E. coli*, *H. pylori*, *K. oxytoca*, *S. typhi*, *S. dysenteriae*, *S. pneumoniae*, *Y. enterocolitica*	*C. difficile* and *L. monocytogenes*
	Second (334–354)	*C. jejuni*, *H. pylori*, MAP, *M. leprae*, *M. tuberculosis*, *S. typhi*.	*C. difficile* and *L. monocytogenes*
	Third (516–530)	None of them	
	Fourth (685–699)	None of them	

### 3.5 Docking and MD virtual screening for analyzing the HLA-epitope complex interactions

**Fig 3:**
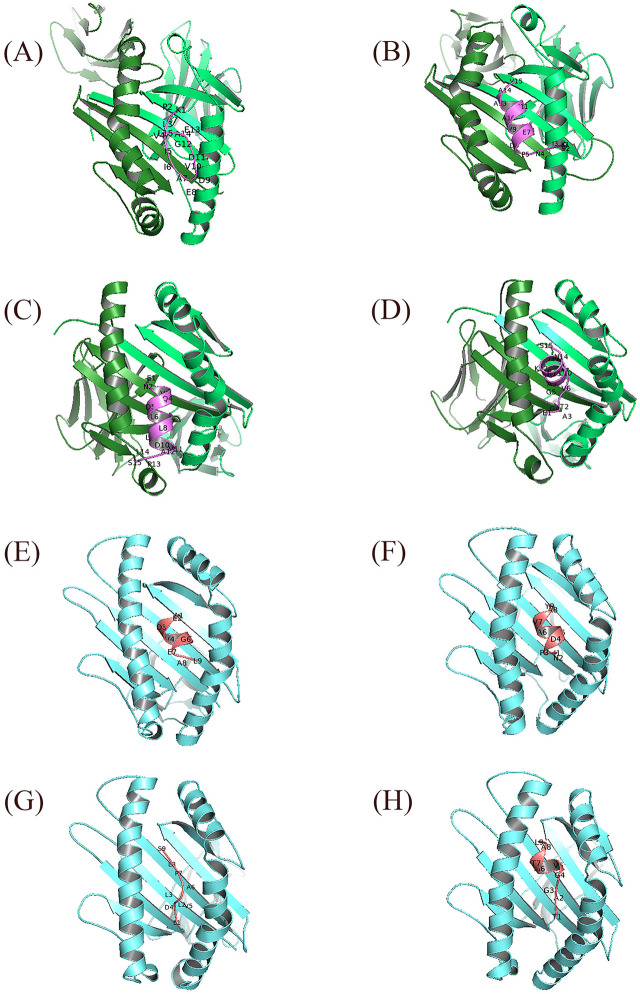
The visualized predicted models of the three-dimensional (3D) structures of MHCs in complex with the four final selected epitopes. Docking depicts the interaction of selected epitopes with HLA in the active site region. This region illustrates the amino acids of the alpha helix of the agretope in the binding groove (detailed in Figs 6−9, based on MD results). Deep salmon and violet colors represent each epitope related to CTL and HTL, respectively. MHC-I and MHC-II receptors are depicted in aquamarine and green (forest color for chain A and lime green for chain B). Options: HLA Class II alleles, (A) HLA-DQ2 (DQ B1*02/ 6U3M- 1.90 Å resolution) (https://www.rcsb.org/structure/6U3M) carrying KPLVIIAEDVDGEAL (HSP 60- from 269 to 283 AA); (B) DRB1*01:01 (7YX9- 1.76 Å resolution) (https://www.rcsb.org/structure/7YX9) carrying KSINPDEAVAYGAAV (HSP 70- from 361 to 375 AA); (C) DRB1*04:01 (4MD4- 1.95 Å resolution) (https://www.rcsb.org/structure/4MD4) carrying ENVQDLLLLDVAPLS (HSP 70- from 386 to 400 AA); (D) DRB1*11:01 (6CPL- 2.45 Å resolution) (https://www.rcsb.org/structure/6CPL) carrying ETAGGVMTALIKRNS (HSP 70- from 404 to 418 AA). HLA Class I allele: (E) HLA-B*44:02 (B44/ 3KPM- 1.60 Å resolution) (https://www.rcsb.org/structure/3KPM) carrying AEDVDGEAL (HSP 60- from 275 to 283 AA); (F) HLA-B*15:01 (B62/ 5TXS- 1.70 Å resolution) (https://www.rcsb.org/structure/5TXS) carrying INPDEAVAY (HSP 70- from 363 to 371 AA); (G) HLA-A*02:01 (6TDS- 1.70 Å resolution) (https://www.rcsb.org/structure/6TDS) carrying LLLDVAPLS (HSP 70- from 392 to 400 AA); (H) HLA-B*14:02 (3BXN- 1.86 Å resolution) (https://www.rcsb.org/structure/3BXN) carrying TAGGVMTAL (HSP 70- from 405 to 413 AA). Abbreviations: HSP, heat shock protein; MHC, major histocompatibility complex; HLA, human leukocyte antigen; CTL, cytotoxic T lymphocyte; HTL, helper T lymphocyte.

The specific affinities and binding patterns of a subset of epitopes and HLA alleles were determined through Molecular docking analysis (Fig 3). The predictive models generated by the LZerD web server algorithm were organized according to the Ranksum score, which is determined by summing the ranks from three individual scores; a lower score suggests a more favorable result. This Ranksum score served as the primary method for ranking the predictive models. When interpreting the results, using the Ranksum score as the basis for model selection is advisable. PDBsum reported the Ramachandran plot statistics of docking complexes in S4 Table and S3 Fig. The optimal Phi and Psi degrees were determined for complex regions, including those classified as most favored, additional allowed, generously allowed, and disallowed.

Our team utilized molecular docking (MD) simulation to examine the structural protein-protein interaction, stability, energy, and functional aspects during various phases of affinity development. We ultimately selected four epitopes that accurately cooperate and interact with immune receptors through careful analysis, as confirmed by both docking and MD analysis. Our system demonstrated sufficient stability for simulation, as shown in the temperature graph analysis presented in S4 Fig. Additionally, S5 and S6 Figs provide comprehensive analyses of pressure and potential energy. The total energy balance following convergence is depicted in the graph found in S7 Fig. We also examined the radius of gyration of HLA-Epitope complexes in S8 and S9 Figs, respectively, to determine the flexibility and dynamic behavior of the simulated complexes. During the simulation, a lower Rg value indicates a more compact and stable protein structure, while higher values of Rg fluctuation indicate a less stable protein.

**Fig 4:**
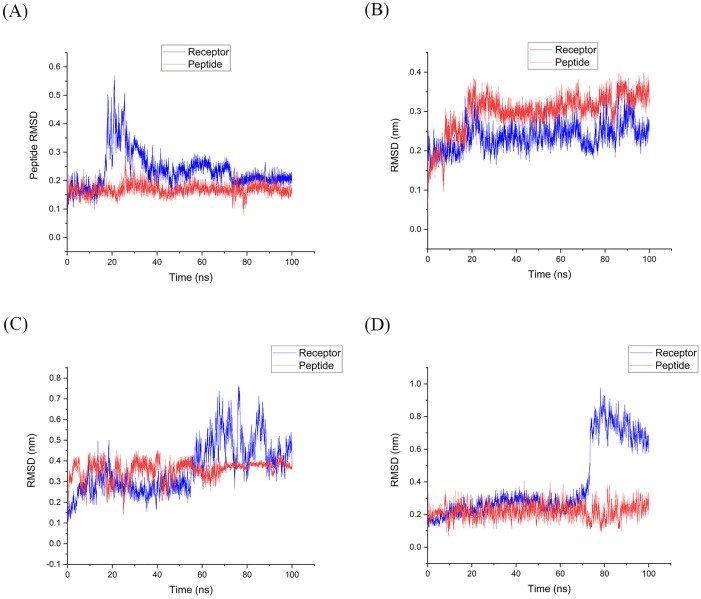
RMSD plot of HLA-epitope complexes backbone in A-D complexes. (A) The complex of HLA DQ2 (DQB1*02) HSP60 selected peptide; (B) The complex of HLA DRB1*11:01 HSP70−6 selected peptide; (C) The complex of HLA DRB1*01:01 HSP70−4 selected peptide; (D) The complex of HLA DRB1*04:01 HSP70−5 peptide chosen. (Receptor: HLA; Ligand: Epitope). The RMSD of the HLA as a receptor in complex A initially increased to around 0.55 nm during the first 20 nanoseconds of simulation. Afterward, it stabilized at 3 angstroms from around 40 nanoseconds until the end of the simulation. During the simulation, the epitope, acting as a ligand, quickly reached equilibrium with minimal fluctuations and an average RMSD of 0.2 nm. For further analysis of this complex, we selected the last 60 nanoseconds of the simulation. Complex B achieved stability between 20 and 100 nanoseconds, with the HLA exhibiting an average RMSD value of 0.3 nm and the epitope having an RMSD value of 0.25 nm. Complex C demonstrated an RMSD value of 0.4 nm, and the receptor achieved relative stability after 60 ns. Throughout the simulation, the RMSD value of the epitope remained stable with low fluctuations, averaging 0.35 nm. In complex D, the HLA remained steady at a distance of 0.2 nm for 80 ns before undergoing a conformational change and stabilizing at 0.6 nm. Meanwhile, the epitope remained consistently stable at a distance of 0.2 nM throughout the simulation. Abbreviations: RMSD, root mean square deviation; MD, molecular dynamics; HLA, human leukocyte antigen.

According to Fig 4, during the simulation, as part of the RMSD analysis in the MD evaluation, the epitope acted as a ligand and quickly reached equilibrium, exhibiting minimal fluctuations and the mentioned average RMSD. We selected the stability state of the simulation for further analysis of each complex. These findings suggested that the HLA-Epitope complexes in this study were robust.

**Fig 5:**
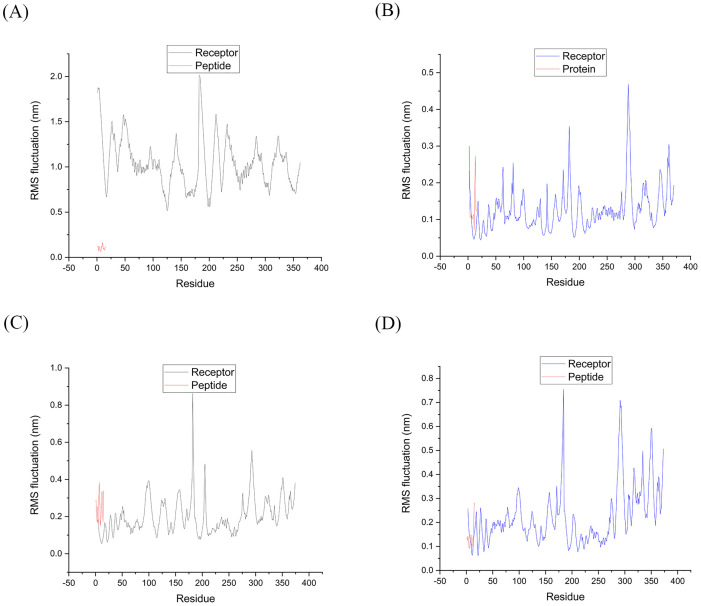
During simulation, the RMSF plots of HLA-Epitope residues in complexes A to D are shown until the system reaches equilibrium. (A) The complex of HLA DQ2 (DQB1*02) HSP60 selected peptide; (B) The complex of HLA DRB1*11:01 HSP70−6 selected peptide; (C) The complex of HLA DRB1*01:01 HSP70−4 selected peptide; (D) The complex of HLA DRB1*04:01 HSP70−5 peptide chosen. (Receptor: HLA; Ligand: Epitope). For complexes A to D, the average RMSF of the protein was 1.04, 0.12, 0.2, and 0.22 nm, respectively. On the other hand, the peptides displayed an average RMSF of 0.1, 0.13, 0.24, and 0.14 nm for the same complexes. Abbreviations: RMSF, root mean square fluctuation; MD, molecular dynamics; HLA, human leukocyte antigen.

According to the results illustrated in Fig 5, the peaks observed in the RMSF plots indicate the areas that experienced the most substantial fluctuation throughout the simulation. It had been established that the N-terminal and C-terminal regions, along with the helices and turns in proteins, displayed the highest degree of structural flexibility. These regions were highly dynamic and accounted for most of the fluctuations in protein structure.

**Fig 6:**
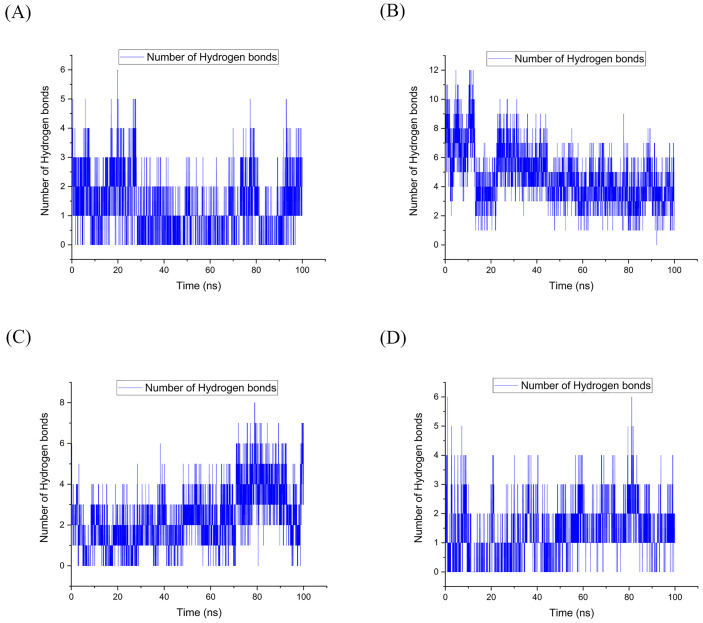
Throughout the MD simulation, multiple hydrogen bonds were observed between the epitope and HLA in complexes A through D. (A) The complex of HLA DQ2 (DQB1*02) HSP60 selected peptide; (B) The complex of HLA DRB1*11:01 HSP70−6 selected peptide; (C) The complex of HLA DRB1*01:01 HSP70−4 selected peptide; (D) The complex of HLA DRB1*04:01 HSP70−5 selected peptide). The number of bonds formed varied, with complex A showing an average of 1.21 bonds (up to 6), complex B exhibiting an average of 4.65 bonds (up to 12), and complex C forming an average of 2.29 bonds (up to 8). Complex D displayed an average of 1.21 bonds (up to 6). Abbreviations: MD, molecular dynamics; HLA, human leukocyte antigen.

According to Fig 6, stable hydrogen bonds between HLA and the epitope were observed in all four complexes during the 100-ns simulation period.

**Fig 7:**
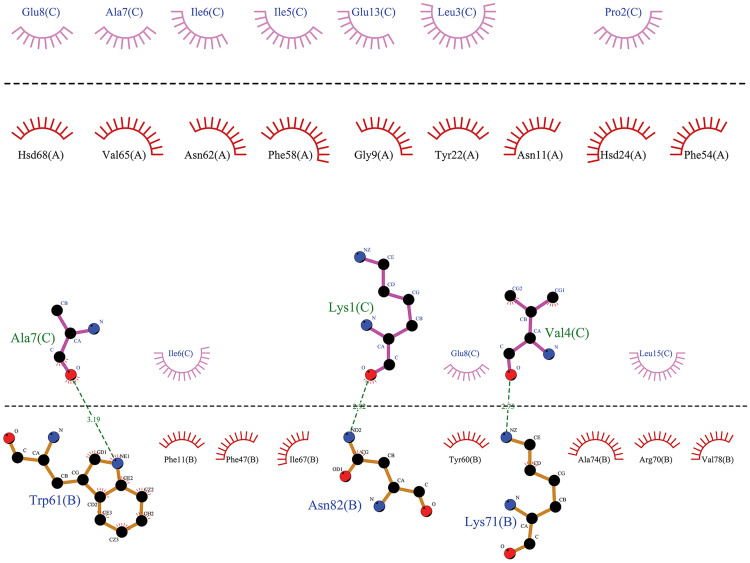
The graphical representation has been provided to illustrate the interaction between the agretope of the epitope (C) as the sequence “KPLVIIAEDVDGEAL” with the HLA binding pockets (chains A and B) in the HLA-DQ2 (DQB1*02)-HSP60 complex. It has been observed that the epitope does not interact with the A chain of the HLA-DQ2 receptor. However, a hydrophobic interaction has been noted in the B chain, centered on the Ala No. 7, Lys No. 1, and Val No. 4 residues from the 15mer sequence of the selected HSP60 epitope.

**Fig 8:**
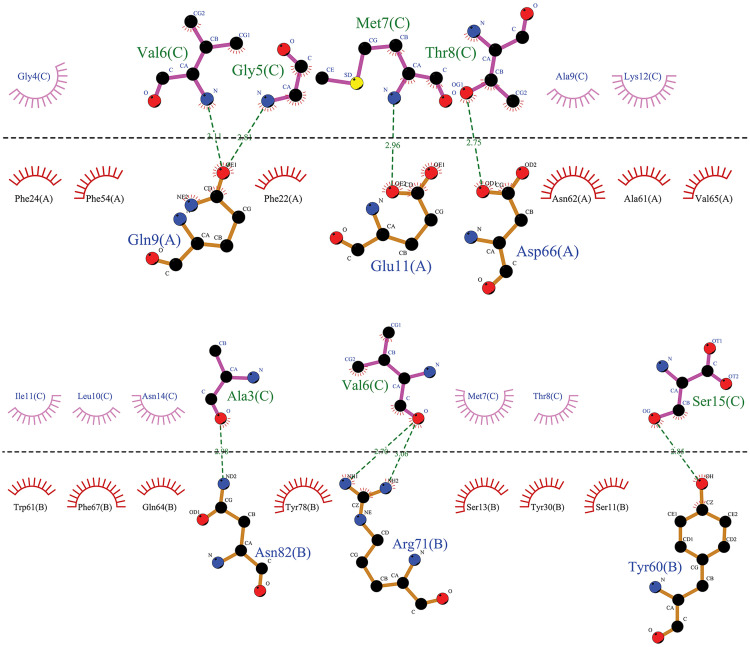
This image depicts the interaction between the agretope of the epitope (C) as the sequence “ETAGGVMTALIKRNS” with residues of the receptor binding pocket of the HLA-DRB1*11:01 (chains A and B) in the HLA-DRB1*11:01-HSP70-6 complex. Notably, an evident hydrophobic interaction exists between specific epitope residues, including Val No. 6, Gly No. 5, Met No. 7, Thr No. 8, and the A chain of the HLA-DRB1*11:01 receptor. Additionally, we can observe a hydrophobic interaction between residues Ala No. 3, Val No. 6, and Ser No. 15 of the 15mer sequence of the sixth selected epitope of HSP70 protein with the B chain of the receptor.

**Fig 9:**
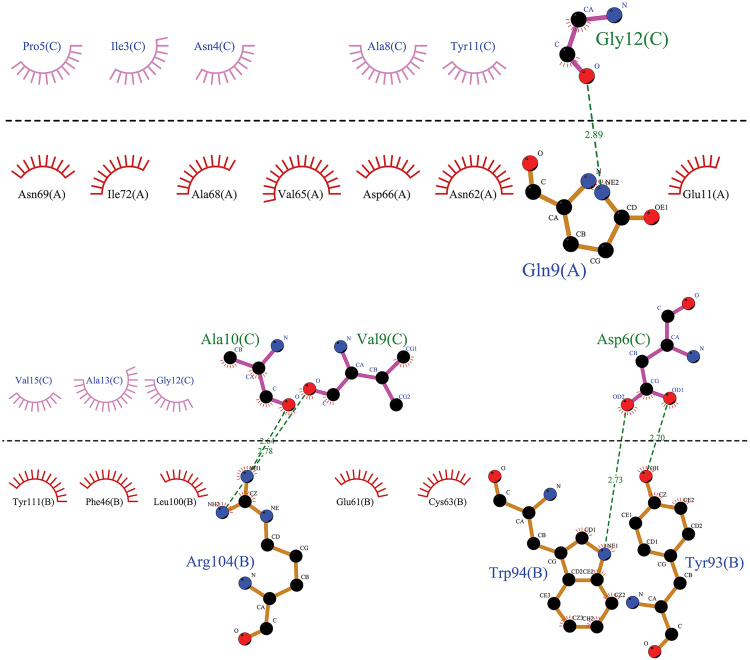
From a two-dimensional perspective, an examination of the interactions between the agretope of the epitope (C) as the sequence “KSINPDEAVAYGAAV” with the residues of the receptor binding pocket (chains A and B) in the HLA-DRB1*01:01-HSP70-4 complex is possible. The data indicate that a hydrophobic interaction is formed between the A chain of the HLA receptor and the epitope residue, facilitated by residue Gly-12 of the epitope. Moreover, a hydrophobic interaction appears between the B chain of the receptor and the fourth selected epitope of the HSP70 protein, and this interaction is facilitated through the cooperation of Ala No. 10, Val No. 9, and Asp No. 6 from the 15mer sequence.

**Fig 10:**
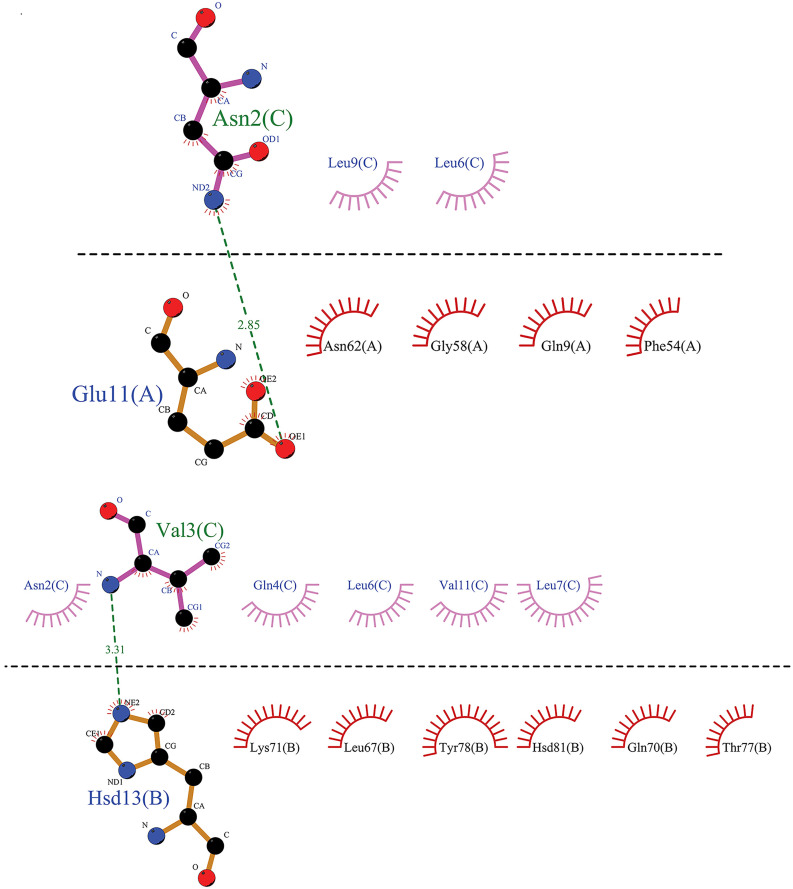
This image depicts the interaction between the agretope of the epitope (C) as the “ENVQDLLLLDVAPLS” sequence, as well as the residues found in the receptor binding pocket (chains A and B) of the HLA-DRB1*04:01-HSP70-5 complex. The figure reveals that only one residue of the fifth selected 15mer epitope of HSP70 has entered into a hydrophobic interaction with each of the receptor chains. Specifically, Asn No. 2 and Val No. 3 interact with the A and B chains of the receptor, respectively.

MD simulation analysis revealed that the HLA DRB1*11:01 HSP70-sixth epitope complex (“ETAGGVMTALIKRNS”) exhibited optimal hydrogen bonding, followed by the HLA DRB1*01:01 HSP70-fourth epitope complex. The HLA DQ2 (DQB1*02) HSP60 epitope complex and the HLA DRB1*04:01 HSP70-fifth epitope complex were equally ranked as the last two (Figs 4–10).

Figs 7–10 highlight the interplay between the agretope of epitopes and the residues of the binding pocket of HLAs that act as receptors as part of secondary outcomes or results. This study included a schematic representation of this interplay to aid comprehension. This region showed the interactive amino acids of the alpha helix of the binding groove.

### 3.6 Host and meta co-expression patterns

Using Pearson’s correlation coefficient, we evaluated the gene expression of the 9 HSPs and 23 highly correlated with IBD-identified genes, as well as the specified number of genes in the metatranscriptome samples among the 13 standard samples, including both types of samples. S2 File Sheet 6 and S4 File Sheet 1 display seven human HSP genes and 86 microbial genes with a Pearson correlation coefficient greater than or equal to 0.9, demonstrating their satisfactory expression in individuals with IBD. All correlation coefficients observed had a p-value of less than or equal to 0.01, indicating a statistically significant level of correlation. Additionally, S2 File Sheet 6 shows that specific human genes are highly correlated with IBD and microbial groups, with a Pearson correlation coefficient of 0.98 or greater in 13 standard samples. The critical point is that the Pearson correlation coefficient of any sample was not smaller than or equal to −0.98. The p-value was less than or equal to 0.01 in all cases, indicating a high level of statistical significance. It should be noted that when using threshold limits of 0.9 and −0.9 to report the results, 4058 gene pairs with acceptable correlation were identified, which, due to their large number, were not reported here. Among these genes, six belonged to the human group, and 140 belonged to the microbial group. In general, it can be stated that both human and microbial genes displayed acceptable expression in IBD.

Furthermore, in S4 File Sheet 2 manifested annotated bacterial transcripts that exhibit a high correlation (Pearson correlation coefficient of 0.8 or greater) with human non-pseudogene HSP transcripts, namely HSPA12A, HSPA12B, HSPA6, and HSPA13 from the HSP70 family, and HSP90B1 from the HSP90 protein family. These HSP transcripts were in common with those from the feature selection process. The greater than 0.9 correlated bacterial transcripts to these 3 HSPs were presented in the previous section in sheet 1 of S4 File. On the other hand, for the 12 other non-pseudogenes not included in the feature selection, no correlations were observed that were greater than 0.9 or smaller than −0.9. A total of 161 meta-transcriptomes with correlations exceeding 0.8 are detailed in S4 File Sheet 3 (all with a p-value of 0.01 or lower). Annotated bacterial transcripts related to folding, chaperone, and stress mechanisms are highlighted in red for easy identification.

During the feature selection and RF process to distinguish between IBD and non-IBD individuals, the analysis of nine host HSP transcripts revealed an interesting finding. We discovered significant correlations between human-specific HSPs and bacterial chaperones, characterized by their respective K numbers. For instance, we identified eight unique chaperone K numbers and folding proteins with a correlation of over 0.9 that are linked to human HSPs commonly associated with RF, such as K03686 (DnaJ, the counterpart of HSP60). Additionally, we identified 18 unique chaperone K numbers and folding proteins with a correlation coefficient of over 0.8 associated with human HSPs common to RF, including K04078 (GroES, as the HSP60 counterpart). Moreover, for human HSPs that are not commonly associated with RF, we identified nine unique chaperone K numbers and folding proteins, with a correlation coefficient of over 0.8, including K03686 (DnaJ, the HSP60 counterpart) and K04078 (GroES, the HSP60 counterpart). After eliminating duplicates, we found 24 unique chaperone K numbers correlated with bacterial chaperones and folding proteins, demonstrating a strong and significant correlation with human HSPs. As an intriguing finding, the genes HSPA12A (mentioned in the RF result) and HSPA5 (not mentioned in the RF), exhibited, strong correlations of above 0.9 and 0.8, respectively, with the bacterial DnaJ (a co-chaperone of DnaK, the bacterial equivalent of HSP70), with statistical significance at p ≤ 0.01 (S4 File, Sheets 1 and 3). Feature selection and RF analysis indicated that HSPA12A can differentiate between individuals with IBD and those without, whereas HSPA5 cannot show this capability. Both genes encoded HSP70 family members [123–127] and showed a significant correlation with bacterial chaperones, particularly DnaJ, as annotated in K03686 which plays a role in protein folding, as supported by information from the KEGG and IGC databases. The KEGG Orthology database was utilized to standardized gene classifications based on function and evolutionary relationships [120,128,129].

## 4 Discussion

Current research suggests that IBD is a complex multifactorial disorder involving immunological, genetic, environmental, microbial interactions and behavioral aspects, as its pathophysiology [6,11,13,47]. This investigation aims to explore the relationship between HLA, the most polymorphic protein [20,23,130], and HSPs, among the most conserved proteins [34,35,40,42], to elucidate their roles in IBD pathogenesis. While certain HLA types, such as HLA-B27, HLA-B51, and HLA-DRB1, have been implicated in IBD, their precise contributions remain unclear [4,5,20,27]. Our primary endpoint revealed meaningful sequence similarity between one epitope of human HSP60 and three epitopes of HSP70 and their bacterial counterparts, supported by MD simulations. The secondary endpoints included identifying residues in the agretope and receptor binding groove of the epitope-HLA complex, as well as bacteria exhibiting considerable similarities to their HSP counterparts and HLA alleles restricting these epitopes. The findings suggest that such epitopes can trigger autoimmune responses in the context of infections via T cells, consistent with the known involvement of CTL in IBD [131].

The interaction between cellular stress responses and gut microbiota is essential for intestinal homeostasis and understanding the pathogenesis of IBD [2,3,10,132]. HSPs play dual roles in IBD pathogenesis. Extracellular HSPs can act as damage-associated molecular patterns (DAMPs) during cellular stress, triggering the inflammatory process observed in IBD. In contrast, intracellular HSPs that evoke the misfolding of specific HLA proteins could contribute to immunomodulation, potentially preventing autoimmune disease [33,34,82,133]. HSP60, a protein located in mitochondria, typically remains hidden on the plasma membrane [38]. HSP60 and HSP70 are highly conserved across species and can induce pro-inflammatory and immunoregulatory processes, acting as molecular adjuvants, enhancing antigen presentation and cytokine production. These processes are linked to the activation of the inflammasome, NF-κB signaling, and reactive oxygen species generation, all of which are involved in the pathogenesis of IBD [34,35,37,41,42,82,133–138].

Studies have explored the role of HSP60 and HSP70 in treating autoimmune conditions such as rheumatoid arthritis, type I diabetes, and IBD due to their potential to induce immunological tolerance [33,34,39]. HSPs can both activate and suppress inflammasomes, indicating the importance of carefully selecting HSP modulators in therapeutic applications. For instance, both the overexpression of HSP70 and the inhibition of HSP90 with geldanamycin effectively halt the activation of the NLRP3 inflammasome due to HSP90 role in folding pro-inflammatory proteins [40,139,140]. According to the findings of this study, a targeted strategy focused on specific epitopes derived from HSPs has the potential to facilitate an understanding of immunopathology and inform future therapeutic investigations. This approach can potentially be more productive and streamlined compared to examining the complete framework of HSPs.

Molecular mimicry and epitope dissemination are recognized mechanisms involved in autoimmunity [2,17,33,38,49]. These concepts have been explored in the context of IBD [2,9,33,49,141], particularly due to the influence of viral proteins on changes in bacterial diversity associated with IBD [49], as well as in other autoimmune disorders related to HSP epitopes [17,38]. Molecular mimicry occurs when antigens derived from a pathogen cross-react with host proteins. This process can activate naive autoreactive T lymphocytes, which recognize both non-self, like infectious agents, and self-epitopes, and potentially initiate autoimmune responses [2,4,14,16,26,36,91,131]. Animal model studies have demonstrated that structural similarity among microbial cross-reactive epitopes can lead to the development of autoimmune diseases [11,14,15,40,43,142,143]. Nevertheless, our immunoinformatic analysis identified specific bacterial HSP epitopes featuring conserved motifs that can induce molecular mimicry, even in the absence of identical amino acid sequences [144–147]. These findings indicate that these epitopes can facilitate comparable synapses between TCRs, peptides, and HLA configurations.

This cross-reactivity supports the activation paradigm of CTLs and HTLs in IBD pathogenesis [2,131,148], as demonstrated in animal models and previous reports. For instance, animal model study showed that introducing HSP60-reactive CD8 + T cells into mice significantly triggered inflammation in the intestinal or throughout the body, depending on the MHC-I context [33,149]. This type of activation, particularly the dysregulation of the TH1 response in the GALT in response to luminal microorganisms and their antigens, was is considered a potential mechanism for IBD pathogenesis [2]. Furthermore, this activation paradigm, where multiple HLA epitopes from different potential target proteins are recognized by various TCRs, is supported by earlier findings [15,48,55,86,131,150–153]. A thorough investigation into the immunogenetic aspects of overlapping CTL and HTL epitopes is essential for understanding cellular immunity [27,79,91]. When these epitopes are presented by MHC-I and MHC-II molecules, they form epitope-HLA complexes that activate and stimulate T cell proliferation. In the case of HTL, this process also activates B cells.

Using computational biology tools, consistent with other studies [17,154], we selected and evaluated thirteen epitopes for their potential to induce autoinflammatory or autoimmune responses, focusing on immunological criteria such as sequence conservation, affinity, immunogenicity, and similarity to human proteins. Our findings highlighted a 15-mer sequence, “KPLVIIAEDVDGEAL,” (positions 269–283 of the human HSP 60 protein), which showed 100% identity with its bacterial counterparts, except for L. monocytogenes, which exhibited 86% similarity. Additionally, other promising epitopes from HSP70 were identified at specific positions, including 15-mer sequences, “KSINPDEAVAYGAAV,” “ENVQDLLLLDVAPLS,” and “ETAGGVMTALIKRNS,” which were aligned with positions 361–375, 386–400, and 404–418 within the human HSP 70 protein, respectively (refer to Table 1 and S11 File). MD simulations indicated stable HLA-epitope complexes, supporting their role in facilitating molecular mimicry and potentially informing future research. The extensive homology between human and bacterial HSP 60 and HSP 70 epitopes across various HLA groups also warrants further research into altered peptide ligands (APLs) [17,33,34,36,37,41,155] and the develop immunomodulating oligonucleotides by mimicking bacterial DNA [156].

Recent research has elucidated the complex interplay between gut microbiota and T lymphocytes, shedding light on the pathogenesis of IBD and potentially paving the way for novel targeted immunopharmacological interventions [2,10,157,158]. Antigen-specific therapies, also known as APLs, show promise in developing innovative treatments [155,159–161], by limiting T-cell activation, modulating immune responses, competing with immunogenic peptide ligands for TCR binding, and inducing low-affinity interactions between HSP-derived epitope-HLA complexes and autoreactive T cells [143,144,162–165]. Ongoing research aims to identify key antigens associated with IBD, with the goal of creating therapies that target these antigens. However, the safety and efficacy of these therapies still require further examination [143,166]. Targeted epitopes offer prospects for antagonistic substances that induce tolerance in autoreactive T cells, thereby potentially immunomodulation, and anergy. This approach could inspire the development of therapeutic monoclonal antibodies, immunomodulatory peptides, and vaccines for autoimmune and auto-inflammatory diseases [26,167]. Additionally, strategies that involve suppressive and exhausted T lymphocytes and modified epitopes could provide new treatment possibilities for IBD [6,131]. In this way, in vitro studies suggest that modified epitopes may effectively induce T-cell exhaustion. Notably, APLs, also known as frameshift or alternative reading frame peptides, have shown potential as neoantigen vaccines in cancer therapy and inflammatory immune diseases. Previous research has also considered oral vaccines based on HSP60 epitopes and APL derived from them. Still, their impact on autoimmune and auto-inflammatory disorders remain important areas for further exploration [155,168–171].

Furthermore, the IFN-γ epitopes were found to upregulate IFNs-related genes and their potential effects on both adaptive and innate immunity, particularly in relation to the MHC-I presentation pathway associated with HSPs and the development of IBD [1,4,9,15,34,55,82,83,151]. It highlights the importance of the immunodominant epitope that stimulates the production of TNF-α in the progression of IBD. This suggests a therapeutic strategy that involves targeting gut flora to enhance the effectiveness of TNF-α inhibitors. The imbalance in intestinal mucosal immunity is identified as a significant factor contributing to IBD, leading to damage to gastrointestinal epithelial cells and disrupted barrier function [1,4,9,151,172]. The study focused on analyzing overlapping epitopes with IFN-γ, as there were no computational tools available for identifying TNF-α-inducing epitopes. It is important to note that specific epitopes may activate other immune cells through complex signaling pathways (Table 1).

An InterPro analysis revealed a conserved domain within HSP60 (amino acids 430–441) that was selected for further study based on the final criteria, including HLA allele density and epitope percentile rank as determined by the IEDB. This led to the selection of epitope 439–456 as the third selective epitope from HSP 60. While this domain did not exhibit considerable similarity across all 13 species analyzed, it did share similarities with two mycobacterial species, *M. leprae* and *Mycobacterium avium paratuberculosis* (*MAP*) (Table 2). Certain regions reported by InterPro were manually excluded from the analysis due to inadequate presentation by diverse HLA alleles, not acceptable IEDB percentile ranks, negative results from the IFN Epitope server, or positive results from ToxinPred.

Our study revealed that sequences of all the studied bacterial HSP 60, HSP 70, and HSP 90 shared remarkable similarities with human HLA-DQA1*05:01-DQB1*02:01-restricted epitopes. The observed similarities extended beyond specific epitopes and affected other regions of bacterial HSPs. This finding suggested potential molecular mimicry and cross-reactivity, especially in genetically predisposed individuals during bacterial infections. These results are consistent with previous research that has linked specific HLA allele epitope complexes to immune-related conditions and autoimmunity through self-antigen mimicry [2,17,144].

Our research contributed to the growing body of evidence implicating specific bacterial species frequently associated with IBD, suggesting their potential to trigger autoimmunity through molecular mimicry [33,50]. While some bacteria such as *Salmonella*, *Campylobacter* [53], *L. monocytogenes* [173,174], *Clostridioides difficile* [15,47,55,162], *E. coli* [9,15,52,55,174,175], *H. pylori* [33,174], *Klebsiella* [9,176], *MAP* [15,33,55,174,177], *S. aureus* [50], *Yersinia* [178,179], *M. paratuberculosis* and *C. jejuni* [180] have been associated with IBD, the precise role of each remains debated, such as MAP, *Streptococcus*, *Clostridia*, *E. coli* [9,173], and *H. pylori* [53,54,181], in the context of IBD [15,152]. At the same time, the infection could trigger an imbalanced immune response against commensal bacteria and molecular mimicry [2,175]. The degree of similarity between bacterial and human proteome (HSPs in our study), particularly in immunogenic epitopes, may underlie these associations and mimic human counterparts. According to some studies, some of the bacterial infections we investigated were not present before the onset of IBD or did not have sufficient time to develop. Subsequently, they could not mimic IBD. In contrast, treatments that suppress the immune system for IBD can raise the chances of reactivating dormant infections [162].

Using both 9-mers and 15-mers enhances immune recognition and is compatible with a wide range of MHC alleles, thereby improving the overall effectiveness of the immune response [182]. Furthermore, pathogenic bacteria can activate the innate immune system, trigger co-stimulatory signals and engaging the acquired immune system. This is particularly relevant in cases of gut dysbiosis and mucosal barrier dysfunction, which can lead to the inflammation commonly associated with IBD [6,35,52,131,183,184]. Future prospective studies, including cohort studies, nested case-control designs, systematic reviews, meta-analyses, and umbrella reviews, should further explore the connection between changes in the gut microbiome, molecular mimicry, and IBD.

The role of HSPs in IBD is a topic of ongoing debate within the scientific community. Exploring HSPs in other autoimmune diseases may uncover mechanisms similar to those in IBD. Research indicates this role supported by HSP-reactive T cells in IBD patients and triggering cross-reaction between bacterial the host’s orthologous HSPs [33–35,82,155,185]. There is no consensus on their specific role in these conditions. Some researchers view HSPs as part of a broader dysbiosis and immune dysregulation, while others believe that certain HSPs directly contribute to the development of the disease. The complexity of IBD, which involves genetic, environmental, and microbial factors, makes it difficult to isolate the specific impact of these proteins. HSP-reactive T cells may have dual effects; they can provide anti-inflammatory responses in some contexts while contributing to inflammation in IBD. This highlights the dual role of HSPs as both protective and pathogenic molecules in different contexts which adds to the challenge of defining their impact on IBD [33–35,82,186].

Our study proposed that molecular mimicry could be a mechanism behind the immunopathogenesis of IBD. The results revealed meaningful sequence similarity between specific epitopes of human HSP60 and HSP70 and their bacterial counterparts. This research identified key interactive residues in epitope-HLA complex. Additionally, HLA alleles restricted the selected epitope were identified, which supported our initial hypothesis through the dry lab immuno-informatics approach. These findings are consistent with previous research conducted through various approaches on molecular mimicry in IBD, such as the work of Cappello et al. (2019), Pahari et al. (2017), Tie et al. (2023), and Brown et al. (2019) [2,9,26,34], and other autoimmune diseases, particularly those associated with Salmonella infection, utilizing the immunoinformatic approach [48]. The similarities in amino acid sequences levels of HSPs between bacteria and humans suggested a potential mechanism for development of IBD through molecular mimicry and subsequent immune activation.

Alongside sequence similarity, our study evaluated the significance of structural similarity, particularly in the case of HSP70 (refer to Fig 2 and S6 File), as well as structural reactions (see Figs 3 and 7–10), highlighting their significance in understanding molecular mimicry. This concentration on both structural and sequence similarities correlated with the findings of Pahari et al. in their 2017 study [26]. Despite the sequential, structural, and functional similarities and the conserved nature of these proteins, variations in specific sequence motifs or regulatory elements can influence their functions and physiology across different species. This understanding is substantial for unraveling cellular signaling and immunological pathways. Recent research has emphasized the importance of structural aspects in the TCR-peptide-MHC (pMHC) interaction and recommends incorporating docking and MD simulations into our analysis [141,150].

In our analysis of the IBDMDB dataset, we uncovered intriguing correlations between specific human HSPs and bacterial chaperones, identified by their K number identifiers. Interestingly, Host and meta-co-expression patterns in the gut environment of IBD individuals indicated the simultaneous presence of HSPs and bacterial chaperones, particularly HSP70 and bacterial DnaJ, both of which belong to the HSP70 family [187–190]. The pronounced correlation between human HSPA12A (r > 0.9) and HSPA5 (r > 0.8), which encode HSP70 in cellular stress responses and bacterial DnaJ, suggests a potential interaction or co-expression pattern in the gut environment under stress, infection, or inflammatory conditions. This co-expression could facilitate cross-reactivity in genetically susceptible individuals, leading to or exacerbating IBD symptoms, where the gut microbiome plays a substantial role in immune system interactions. Bacterial HSP70 and their similarity to human HSP70 proteins might trigger an immune response that cross-reacts with human proteins, potentially leading to autoimmune responses and inflammatory conditions like IBD. Furthermore, our findings built upon and extended the existing body of research investigating bacterial HSPs as potential triggers of molecular mimicry in IBD. [26,33,34,36,38–41,43,133,137,138,153,186].

Additionally, our study of transcriptomes revealed correlations between host HSPs and those of gut bacteria, along with other bacterial genes that are not classified as HSPs, such as proteases and chemotaxis as motility-related genes, provide intriguing insights into the etiology of IBD, as revealed by the IBDMDB database. These findings may suggest a potential convergence in stress response pathways between the host and the microbiome, highlighting the need for further investigation into these interactions and their implications for the pathogenesis of IBD. This added another layer of complexity to this picture, suggesting that molecular mimicry in IBD may involve HSP-HSP interactions and other bacterial proteins that resemble chaperones in structure or function.

The complexity of IBD itself, coupled with the intricacies of its pathogenesis, methodological variations in research studies, and differences in patient populations, often leads to disagreements about the disease. IBD is characterized by a highly personalized pattern of symptoms, triggers, and treatment responses, which makes it challenging to pinpoint the exact role of specific bacteria and their interactions with T-cells across all IBD cases. Furthermore, variations in the technology used to study the microbiome, study design, sample size, and patient demographics can result in different interpretations of data. While there is consensus on the importance of gut microbiota in IBD, the specific contributions of individual bacterial species and their mechanisms remain areas of active research and debate. Some studies suggest that particular pathobionts may exacerbate IBD, while others argue that their presence is a secondary effect of the altered gut environment in IBD patients [2,3,6–8,10,13,52,131,191]. The existing literature reveals conflicting findings regarding the role of HSPs in IBD, indicating a need for more focused research on their potential causative and protective roles. Ongoing targeted studies are necessary to understand the specific HSP epitopes interact with HLAs, as well as the potential of HSPs as biomarkers and therapeutic targets in IBD. From this perspective, future studies, particularly those employing longitudinal designs, high-throughput sequencing technologies, and metagenomics, are crucial for unraveling the complex interplay between the gut microbiota, host immune responses, and HSPs in IBD. This research will further our understanding of the disease and pave the way for innovative therapeutic strategies that target the gut microbiome and these molecular patterns.

The significance of our results lies in the molecular mechanism of immunopathogenesis in IBD. We focused on specific bacterial HSPs, their derived particular epitopes that elicited autoreactive T cells, and residues involved in epitope-HLA complexes. Our findings highlighted the complex interactions between bacterial HSPs in IBD immunopathogenesis through molecular mimicry.

However, our study has several limitations that warrant consideration. First, wet lab validation is needed to confirm the precise epitopes implicated in molecular mimicry in IBD immunopathogenesis, a factor that future research will consider when designing studies to establish definitive causality. In the future, it may be beneficial to clone these specific epitopes and study them in animal models to gain a deeper understanding of how they trigger T-cell-mediated autoinflammatory responses during infections in IBD. Additionally, we employed an exploratory and preliminary approach rather than a confirmatory one. While our study did not include wet lab validation, we acknowledged that it laid the groundwork for future experimental studies that can build on our findings. Additionally, our current work has identified potential targets for further experimental investigation [26,44]. A second limitation was that focusing on structural considerations in the TCR-pMHC triad was required to understand their relationship comprehensively. Third, fourth, and fifth, this study is limited by the lack of computational resources for TNF-α-inducing epitope analysis and by not addressing B-cell interactions or non-bacterial microbes. Sixth, the study’s almost purely immunological perspective is also a limitation. A combination view for therapeutic targets can be more effective [192]. Seventh, when evaluating the host and meta-expression patterns, it’s essential to consider the various types of relationships between gene expression levels. While the Pearson correlation is useful for measuring strong linear relationships, it may not fully capture complex non-linear interactions. Although a strong linear relationship was considered in our study, future studies are recommended to incorporate methods such as Spearman’s rank correlation, which is less sensitive to outliers and can assess non-linear and monotonic relationships more effectively [193,194]. Additionally, using regression models can provide valuable insights into the specific nature of the relationship between HSP expression levels and other genes [195]. Employing partial correlation analysis is also another beneficial tool [196]. Mutual information (MI) is another helpful measure that captures both linear and nonlinear relationships; however, it can be computationally expensive for large datasets [194,197]. By combining these analytical techniques, a more comprehensive assessment of HSP co-expression and other transcriptomes within the IBDMDB dataset can be achieved, taking into account the complexity of these relationships. Lastly, the limitation was that the IBDMDB database represents a specific subset of IBD patients. Therefore, our co-expression findings may not be generalizable to all individuals with the disease.

Despite these limitations, our study has provided considerable insights into our primary and secondary outcomes. Further in vivo and clinical studies are needed to validate these findings and clarify the mechanisms of molecular mimicry in IBD and the exact immunopathogenesis.

While molecular mimicry is still a viable hypothesis for immunopathogenesis of IBD, alternative mechanisms, such as bystander activation and epitope spreading, may also play considerable role. Our study indirectly touched upon genetic predisposition through HLA evaluation and environmental triggers by analyzing microbiota dysbiosis and the presence of specific pathogenic bacteria. However, future research should thoroughly investigate these alternative mechanisms in conjunction lifestyle factors, gut barrier dysfunction, and the variability in host responses to promote a comprehensive understanding of IBD pathogenesis. Moreover, it is essential for future studies to recognize the heterogeneity of IBD and avoid generalizing findings across its entire spectrum. Evaluating whether specific bacterial species and HSP epitopes are more prevalent in one subtype of IBD compared to another could provide deeper into the specific roles of molecular mimicry in different forms of IBD. Our study primarily focused on pathogenic bacteria associated with IBD. Nevertheless, future research should go beyond examining individual bacterial species and conduct a detailed analysis of the broader microbial ecosystem and the complex interactions within the gut microbiota. Investigating whether dysbiosis promotes the proliferation of specific bacterial species that contain HSPs may offer valuable insights into the role of these proteins in IBD.

Furthermore, evaluating whether pathogenic or commensal bacteria produce the identified HSPs is crucial, as this significantly affects the immune response in IBD. In our study, we utilized computational immunology methods and analyzed secondary data from the IBDMDB database to support our approach. While these methods provided valuable insights, they were not sufficient to fully explain the complexities of the immune system in pathogenesis of IBD. Therefore, it is essential to validate our findings through experimental studies, including in vivo and in vitro assays such as T-cell assays, cytokine assays, and animal models. Future research efforts should prioritize these experimental validations to strengthen the conclusions derived from our computational models. Our study also used the IBDMDB database to explore the relationship between human and bacterial HSPs in cases of IBD. To enhance the clinical relevance of our findings, future research should involve more direct clinical correlations and patient-derived data, including the analysis of the gut microbiota in IBD patients for the presence of specific bacterial species and their associated HSPs. Correlating these findings with clinical outcomes, such as disease severity and treatment response, could provide actionable insights. Additionally, future investigation should explore potential therapeutic strategies, such as immunomodulation, vaccination against specific bacterial strains, or probiotics, aimed at targeting microbial HSPs without harming healthy human cells, especially since many HSPs are highly conserved proteins. Ethical and safety considerations should be addressed in subsequent studies. Furthermore, examining HLA polymorphism and incorporate HLA typing in both computational and experimental contexts to determine whether specific HLA alleles are more likely to present bacterial HSPs, which may contribute to a better understanding of susceptibility to IBD and assist in developing personalized treatment plans.

Moreover, the relationship between bacteria and IBD extends beyond the mentioned concept. Recent research suggests that utilizing in-silico methods to investigate anti-inflammatory commensal bacteria such as *Faecalibacterium prausnitzii* may be a promising approach for treating IBD [9,39,46,163,198–202]. Identifying the primary target antigens may enhance our understanding of tolerance to various antigen molecules. This approach aligns with recent studies examining epitope-HLA interactions in the context of auto-inflammatory diseases [4,17,26,36–38,48,144]. Furthermore, the presence of inflammation-related autoimmune diseases, such as multiple sclerosis (MS) and IBD, suggests a potential shared genetic background or similar microbial influences [5,203,204]. This connection raises concerns about an increased risk of colorectal cancer [33,158,167,184,205,206], as well as other autoimmune disorders, including erythema nodosum [5,203], immune thrombocytopenic purpura (ITP) [207], primary sclerosing cholangitis [45,203], and also has implications for coronavirus disease 2019 (COVID-19) [208]. Consequently, the findings of this study may have implications for various conditions associated with the immune system, including auto-inflammatory and autoimmune disorders, as well as IBD.

Understanding these mechanisms may inform future research and therapeutic strategies targeting the immune-microbial interface in IBD [15,28,32,209–211]. Despite challenges in establishing direct causality, molecular mimicry has been increasingly recognized as a pivotal factor in the etiology of autoimmune diseases, based on our current understanding. The pathogenesis of IBD likely involves a bidirectional interplay between inflammation and microbiota dysbiosis, contributing to the disease’s progression and chronicity [2,3]. Advancements in metagenomics and transcriptomics offer promising avenues for high-throughput exploration of this hypothesis, facilitating the examination of gene-disease pathway linkages, differential gene expression (DGE), gene co-expression networks, gene ontology, and protein-protein interactions. Such studies are instrumental in identifying significant antisense or small molecules that could revolutionize treatment approaches and diagnostic procedures, serving as valuable biomarkers [20,27,192,212,213].

Additionally, exploring diverse perspectives, such as interspecies microbial communication and interactions between host microbes and bioactive molecules from gut-derived microorganisms, will enhance our understanding of the microenvironment’s influence on IBD [214–216]. A comprehensive evaluation of the genetic and environmental factors contributing to IBD pathogenesis is essential for a deeper understanding of the disease [1,22,217,218].

## 5 Conclusion

In summary, our dry lab findings suggest that molecular mimicry, where bacterial HLA-restricted immunodominant epitopes of HSPs resemble those of the host, represents a compelling mechanism for the immunological connection between bacteria and prevalent HLA alleles in IBD pathogenesis. This approach bolstered and provided more evidence for the proposed molecular mimicry hypothesis, suggesting structure and sequence similarities between antigens of gut-derived microorganisms and host HLA epitopes that may induce cross-reactivity. Identifying these target proteins and their putative T-cell epitopes could catalyze extensive research into the molecular underpinnings of auto-inflammatory responses during infection. Moreover, this understanding may offer a framework for predicting the complex pathogenesis of IBD, thereby enhancing our ability to suggest novel interventions. The structural resemblances can be leveraged to develop targeted therapies that modulate the immune response and mitigate tissue damage.

## Supporting information

S1 File Paint epitopic area. This Excel file illustrates epitopic area painting to select the final epitopes through various important filters per HSP60, 70, and 90 in separate sheets.
(XLSX)

S2 File IBDMDB samples analysis. The analysis of IBDMDB samples is presented in this file as follows: Sheet 1: Features selected for human HSPs and meta transcripts. Sheet 2: Selected host and meta transcriptomic samples. Sheets 3 and 4: Transcriptomic IDs for non-IBD hosts and meta transcriptomics, respectively. Sheet 5: Displays 13 common samples. Sheet 6: Contains the final selected host and meta-transcriptomics.
(XLSX)

S3 File IBDMDB-selected gene for random forest. This file represents host HSPs’ selected gene from IBDMDB for random forest in sheet one and the highly correlated gene with IBD in sheet two.
(XLSX)

S4 File Correlated host-meta transcript. Correlated host-meta transcript, greater than 0.9 and 0.8, is presented in this file.
(XLSX)

S5 File PsiBLAST. This file displays PsiBLAST and BLAST Tree for HSP60 in the first and third iterations, HSP70 in the first and fifth iterations, and HSP90.
(XLSX)

S6 File Structural alignment. This file showcases different aspects of structural alignment between human HSPs and their bacterial counterparts.
(XLSX)

S7 File CTL epitopes. The Excel file illustrates the characteristics of CTL epitopes related to HSPs.
(XLSX)

S8 File HTL-HSP60 epitopes. After applying immunological filters, overlapping HTL and CTL epitopes of HSP 60 restricted by HLA alleles are identified in this file. Potential immunodominant overlapping HTL epitopes are found in sheet one, while HSP60-MHC-II non-overlapping HTL epitopes are located in sheet two.
(XLSX)

S9 File HTL-HSP70-epitopes. HSP 70-overlapping HTL and CTL epitope mapping restricted by HLA alleles can be found in sheet 1, while HSP70-MHC-II non-overlapping HTL epitopes are listed in sheet 2, based on IEDB. The application of immunological filters is also represented.
(XLSX)

S10 File HTL-HSP90 epitopes. This file outlines the selection of HSP 90-overlapping HTL and CTL epitopes restricted by HLA alleles on sheet 1, and HSP90-MHC-II alleles-non-overlapping HTL epitopes on sheet 2 through applied immunological filters.
(XLSX)

S11 File Bacterial-pairwise alignment. This document presents the final selection of epitopes based on a comparative analysis of epitopic regions. It shows sequence similarity between human HSPs and their bacterial counterparts in the restricted epitopes recognized by different HLA populations. Furthermore, the results suggest that specific HLA alleles are the most restrictive for the majority of putative immunodominant peptides that resemble the epitopes found on human HSPs.
(XLSX)

## References

[1] Stange EF. Current and future aspects of IBD research and treatment: the 2022 perspective. Front Gastroenterol. 2022;1. doi: 10.3389/fgstr.2022.914371 PMC12952443 41822078

[2] Brown EM, Kenny DJ, Xavier RJ. Gut microbiota regulation of T cells during inflammation and autoimmunity. Annu Rev Immunol. 2019;37:599–624. doi: 10.1146/annurev-immunol-042718-041841 31026411

[3] Ning L, Zhou Y-L, Sun H, Zhang Y, Shen C, Wang Z, et al. Microbiome and metabolome features in inflammatory bowel disease via multi-omics integration analyses across cohorts. Nat Commun. 2023;14(1):7135. doi: 10.1038/s41467-023-42788-0 37932270 PMC10628233

[4] Verma A, Sharda S, Rathi B, Somvanshi P, Pandey BD. Elucidating potential molecular signatures through host-microbe interactions for reactive arthritis and inflammatory bowel disease using combinatorial approach. Sci Rep. 2020;10(1):15131. doi: 10.1038/s41598-020-71674-8 32934294 PMC7492238

[5] Karlinger K, Györke T, Makö E, Mester A, Tarján Z. The epidemiology and the pathogenesis of inflammatory bowel disease. Eur J Radiol. 2000;35(3):154–67. doi: 10.1016/s0720-048x(00)00238-2 11000558

[6] Graham DB, Xavier RJ. Pathway paradigms revealed from the genetics of inflammatory bowel disease. Nature. 2020;578(7796):527–39. doi: 10.1038/s41586-020-2025-2 32103191 PMC7871366

[7] Berg G, Rybakova D, Fischer D, Cernava T, Vergès M-CC, Charles T, et al. Microbiome definition re-visited: old concepts and new challenges. Microbiome. 2020;8(1):103. doi: 10.1186/s40168-020-00875-0 32605663 PMC7329523

[8] Alam MT, Amos GCA, Murphy ARJ, Murch S, Wellington EMH, Arasaradnam RP. Microbial imbalance in inflammatory bowel disease patients at different taxonomic levels. Gut Pathog. 2020;12:1. doi: 10.1186/s13099-019-0341-6 31911822 PMC6942256

[9] Tie Y, Huang Y, Chen R, Li L, Chen M, Zhang S. Current insights on the roles of gut microbiota in inflammatory bowel disease-associated extra-intestinal manifestations: pathophysiology and therapeutic targets. Gut Microbes. 2023;15(2):2265028. doi: 10.1080/19490976.2023.2265028 37822139 PMC10572083

[10] Qiu P, Ishimoto T, Fu L, Zhang J, Zhang Z, Liu Y. The gut microbiota in inflammatory bowel disease. Front Cell Infect Microbiol. 2022;12:733992. doi: 10.3389/fcimb.2022.733992 35273921 PMC8902753

[11] Hansen R, Thomson JM, El-Omar EM, Hold GL. The role of infection in the aetiology of inflammatory bowel disease. J Gastroenterol. 2010;45(3):266–76. doi: 10.1007/s00535-009-0191-y 20076977 PMC7087869

[12] Terato K, Do CT, Shionoya H. Slipping through the cracks: linking low immune function and intestinal bacterial imbalance to the etiology of rheumatoid arthritis. Autoimmune Dis. 2015;2015:636207. doi: 10.1155/2015/636207 25861466 PMC4377354

[13] Yau YY, Wasinger VC, Hirten RP, Chuang E, Huntsman M, Stylli J, et al. Current trends in IBD-development of mucosal-based biomarkers and a novel minimally invasive recoverable sampling system. Inflamm Bowel Dis. 2021;27(Suppl 2):S17–24. doi: 10.1093/ibd/izab179 34791290 PMC9214562

[14] Battaglia M, Garrett-Sinha LA. Bacterial infections in lupus: roles in promoting immune activation and in pathogenesis of the disease. J Transl Autoimmun. 2020;4:100078. doi: 10.1016/j.jtauto.2020.100078 33490939 PMC7804979

[15] Sartor RB. Microbial influences in inflammatory bowel diseases. Gastroenterology. 2008;134(2):577–94. doi: 10.1053/j.gastro.2007.11.059 18242222

[16] Kivity S, Agmon-Levin N, Blank M, Shoenfeld Y. Infections and autoimmunity--friends or foes? Trends Immunol. 2009;30(8):409–14. doi: 10.1016/j.it.2009.05.005 19643667

[17] Chodisetti SB, Rai PK, Gowthaman U, Pahari S, Agrewala JN. Potential T cell epitopes of Mycobacterium tuberculosis that can instigate molecular mimicry against host: implications in autoimmune pathogenesis. BMC Immunol. 2012;13:13. doi: 10.1186/1471-2172-13-13 22435930 PMC3359254

[18] Gudeta AN, Andrén Aronsson C, Binagdie BB, Girma A, Agardh D. Incidence of celiac disease autoimmunity and associations with maternal tuberculosis and pediatric Helicobacter pylori infections in 4-year-old Ethiopian children followed up in an HLA genotyped birth cohort. Front Pediatr. 2022;10:999287. doi: 10.3389/fped.2022.999287 36389354 PMC9644195

[19] Bouziat R, Hinterleitner R, Brown JJ, Stencel-Baerenwald JE, Ikizler M, Mayassi T, et al. Reovirus infection triggers inflammatory responses to dietary antigens and development of celiac disease. Science. 2017;356(6333):44–50. doi: 10.1126/science.aah5298 28386004 PMC5506690

[20] Ahmad T, Marshall S-E, Jewell D. Genetics of inflammatory bowel disease: the role of the HLA complex. World J Gastroenterol. 2006;12(23):3628–35. doi: 10.3748/wjg.v12.i23.3628 16773677 PMC4087453

[21] Goyette P, Boucher G, Mallon D, Ellinghaus E, Jostins L, Huang H, et al. High-density mapping of the MHC identifies a shared role for HLA-DRB1*01:03 in inflammatory bowel diseases and heterozygous advantage in ulcerative colitis. Nat Genet. 2015;47(2):172–9. doi: 10.1038/ng.3176 25559196 PMC4310771

[22] Jin X-Y, Li D-D, Quan W, Chao Y, Zhang B. Leaky gut, circulating immune complexes, arthralgia, and arthritis in IBD: coincidence or inevitability? Front Immunol. 2024;15:1347901. doi: 10.3389/fimmu.2024.1347901 38571963 PMC10987687

[23] Pagliuca S, Gurnari C, Rubio MT, Visconte V, Lenz TL. Individual HLA heterogeneity and its implications for cellular immune evasion in cancer and beyond. Front Immunol. 2022;13:944872. doi: 10.3389/fimmu.2022.944872 36131910 PMC9483928

[24] Kovvali G, Das KM. Molecular mimicry may contribute to pathogenesis of ulcerative colitis. FEBS Lett. 2005;579(11):2261–6. doi: 10.1016/j.febslet.2005.02.073 15848155

[25] Thaper D, Prabha V. Molecular mimicry: unravelling the role of autoantibodies in autoimmune diseases and infertility. In: Sobti RC, Sobti A, eds. Biomedical translational research. Springer Nature Singapore; 2022: 395–406. doi: 10.1007/978-981-16-4345-3_24

[26] Pahari S, Chatterjee D, Negi S, Kaur J, Singh B, Agrewala JN. Morbid sequences suggest molecular mimicry between microbial peptides and self-antigens: a possibility of inciting autoimmunity. Front Microbiol. 2017;8:1938. doi: 10.3389/fmicb.2017.01938 29062305 PMC5640720

[27] Muro M, López-Hernández R, Mrowiec A. Immunogenetic biomarkers in inflammatory bowel diseases: role of the IBD3 region. World J Gastroenterol. 2014;20(41):15037–48. doi: 10.3748/wjg.v20.i41.15037 25386052 PMC4223237

[28] Alvarez-Navarro C, Cragnolini JJ, Dos Santos HG, Barnea E, Admon A, Morreale A, et al. Novel HLA-B27-restricted epitopes from Chlamydia trachomatis generated upon endogenous processing of bacterial proteins suggest a role of molecular mimicry in reactive arthritis. J Biol Chem. 2013;288(36):25810–25. doi: 10.1074/jbc.M113.493247 23867464 PMC3764788

[29] Wucherpfennig KW. Structural basis of molecular mimicry. J Autoimmun. 2001;16(3):293–302. doi: 10.1006/jaut.2000.0499 11334495

[30] Crux NB, Elahi S. Human Leukocyte Antigen (HLA) and immune regulation: how do classical and non-classical HLA alleles modulate immune response to human immunodeficiency virus and hepatitis C virus infections? Front Immunol. 2017;8:832. doi: 10.3389/fimmu.2017.00832 28769934 PMC5513977

[31] Bodis G, Toth V, Schwarting A. Role of Human Leukocyte Antigens (HLA) in autoimmune diseases. Rheumatol Ther. 2018;5(1):5–20. doi: 10.1007/s40744-018-0100-z 29516402 PMC5935613

[32] Fourneau J-M, Bach J-M, van Endert PM, Bach J-F. The elusive case for a role of mimicry in autoimmune diseases. Mol Immunol. 2004;40(14–15):1095–102. doi: 10.1016/j.molimm.2003.11.011 15036914

[33] Hoter A, Naim HY. The functions and therapeutic potential of heat shock proteins in inflammatory bowel disease-an update. Int J Mol Sci. 2019;20(21):5331. doi: 10.3390/ijms20215331 31717769 PMC6862201

[34] Cappello F, Mazzola M, Jurjus A, Zeenny M-N, Jurjus R, Carini F, et al. Hsp60 as a novel target in IBD management: a prospect. Front Pharmacol. 2019;10:26. doi: 10.3389/fphar.2019.00026 30800066 PMC6376446

[35] Routsias JG, Tzioufas AG. The role of chaperone proteins in autoimmunity. Ann N Y Acad Sci. 2006;1088:52–64. doi: 10.1196/annals.1366.029 17192556

[36] Ansari Qeshmi S, Dabbagh F, Borhani Haghighi A, Ghasemi Y. Bioinformatics evaluation of the possibility of heat shock proteins as autoantigens in multiple sclerosis based on molecular mimicry hypothesis. J Neuroimmunol. 2016;295–296:100–21. doi: 10.1016/j.jneuroim.2016.03.018 27235356

[37] Ghasemi Y, Dabbagh F, Rasoul-Amini S, Borhani Haghighi A, Morowvat MH. The possible role of HSPs on Behçet’s disease: a bioinformatic approach. Comput Biol Med. 2012;42(11):1079–85. doi: 10.1016/j.compbiomed.2012.08.009 23036375

[38] Lucchese G, Flöel A. SARS-CoV-2 and Guillain-Barré syndrome: molecular mimicry with human heat shock proteins as potential pathogenic mechanism. Cell Stress Chaperones. 2020;25(5):731–5. doi: 10.1007/s12192-020-01145-6 32729001 PMC7387880

[39] Jansen MAA, Spiering R, Broere F, van Laar JM, Isaacs JD, van Eden W, et al. Targeting of tolerogenic dendritic cells towards heat-shock proteins: a novel therapeutic strategy for autoimmune diseases?. Immunology. 2018;153(1):51–9. doi: 10.1111/imm.12811 28804903 PMC5721256

[40] Tukaj S. Heat Shock Protein 70 as a double agent acting inside and outside the cell: insights into autoimmunity. Int J Mol Sci. 2020;21(15):5298. doi: 10.3390/ijms21155298 32722570 PMC7432326

[41] Singh MK, Shin Y, Ju S, Han S, Choe W, Yoon K-S, et al. Heat shock response and heat shock proteins: current understanding and future opportunities in human diseases. Int J Mol Sci. 2024;25(8):4209. doi: 10.3390/ijms25084209 38673794 PMC11050489

[42] Sadat A, Tiwari S, Sunidhi S, Chaphalkar A, Kochar M, Ali M, et al. Conserved and divergent chaperoning effects of Hsp60/10 chaperonins on protein folding landscapes. Proc Natl Acad Sci U S A. 2022;119(18):e2118465119. doi: 10.1073/pnas.2118465119 35486698 PMC9170145

[43] Mansilla MJ, Montalban X, Espejo C. Heat shock protein 70: roles in multiple sclerosis. Mol Med. 2012;18(1):1018–28. doi: 10.2119/molmed.2012.00119 22669475 PMC3459486

[44] Garg A, Kumari B, Kumar R, Kumar M. miPepBase: a database of experimentally verified peptides involved in molecular mimicry. Front Microbiol. 2017;8:2053. doi: 10.3389/fmicb.2017.02053 29109711 PMC5660332

[45] Di Giorgio A, Vergani D, Mieli-Vergani G. Cutting edge issues in juvenile sclerosing cholangitis. Dig Liver Dis. 2022;54(4):417–27. doi: 10.1016/j.dld.2021.06.028 34289942

[46] Huang X, Fan X, Ying J, Chen S. Emerging trends and research foci in gastrointestinal microbiome. J Transl Med. 2019;17(1):67. doi: 10.1186/s12967-019-1810-x 30819194 PMC6396506

[47] Lucas López R, Grande Burgos MJ, Gálvez A, Pérez Pulido R. The human gastrointestinal tract and oral microbiota in inflammatory bowel disease: a state of the science review. APMIS. 2017;125(1):3–10. doi: 10.1111/apm.12609 27704622

[48] Rahman N, Begum S, Khan A, Afridi SG, Khayam Sahibzada MU, Atwah B, et al. An insight in Salmonella typhi associated autoimmunity candidates’ prediction by molecular mimicry. Comput Biol Med. 2022;148:105865. doi: 10.1016/j.compbiomed.2022.105865 35843194

[49] Errico C, Massimino L, Facoetti A, Nicolò S, Cagliani S, Spanò S, et al. P057 caudovirales may trigger molecular mimicry mechanisms in Crohn’s disease. J Crohn’s Colitis. 2024;18(Supplement_1):i321. doi: 10.1093/ecco-jcc/jjad212.0187

[50] Padoan A, Musso G, Contran N, Basso D. Inflammation, autoinflammation and autoimmunity in inflammatory bowel diseases. Curr Issues Mol Biol. 2023;45(7):5534–57. doi: 10.3390/cimb45070350 37504266 PMC10378236

[51] Aldars-García L, Chaparro M, Gisbert JP. Systematic review: the gut microbiome and its potential clinical application in inflammatory bowel disease. Microorganisms. 2021;9(5):977. doi: 10.3390/microorganisms9050977 33946482 PMC8147118

[52] Lee M, Chang EB. Inflammatory Bowel Diseases (IBD) and the microbiome-searching the crime scene for clues. Gastroenterology. 2021;160(2):524–37. doi: 10.1053/j.gastro.2020.09.056 33253681 PMC8098834

[53] Axelrad JE, Cadwell KH, Colombel J-F, Shah SC. Systematic review: gastrointestinal infection and incident inflammatory bowel disease. Aliment Pharmacol Ther. 2020;51(12):1222–32. doi: 10.1111/apt.15770 32372471 PMC7354095

[54] Castaño-Rodríguez N, Kaakoush NO, Lee WS, Mitchell HM. Dual role of helicobacter and campylobacter species in IBD: a systematic review and meta-analysis. Gut. 2017;66(2):235–49. doi: 10.1136/gutjnl-2015-310545 26508508

[55] Ni J, Wu GD, Albenberg L, Tomov VT. Gut microbiota and IBD: causation or correlation? Nat Rev Gastroenterol Hepatol. 2017;14(10):573–84. doi: 10.1038/nrgastro.2017.88 28743984 PMC5880536

[56] Baumgart DC. Crohn’s disease and ulcerative colitis: from epidemiology and immunobiology to a rational diagnostic and therapeutic approach. Cham: Springer International Publishing; 2017. doi: 10.1007/978-3-319-33703-6

[57] Di Tommaso P, Moretti S, Xenarios I, Orobitg M, Montanyola A, Chang J-M, et al. T-Coffee: a web server for the multiple sequence alignment of protein and RNA sequences using structural information and homology extension. Nucleic Acids Res. 2011;39(Web Server issue):W13–7. doi: 10.1093/nar/gkr245 21558174 PMC3125728

[58] Altschul SF, Wootton JC, Gertz EM, Agarwala R, Morgulis A, Schäffer AA, et al. Protein database searches using compositionally adjusted substitution matrices. FEBS J. 2005;272(20):5101–9. doi: 10.1111/j.1742-4658.2005.04945.x 16218944 PMC1343503

[59] Doytchinova IA, Flower DR. Identifying candidate subunit vaccines using an alignment-independent method based on principal amino acid properties. Vaccine. 2007;25(5):856–66. doi: 10.1016/j.vaccine.2006.09.032 17045707

[60] Doytchinova IA, Flower DR. VaxiJen: a server for prediction of protective antigens, tumour antigens and subunit vaccines. BMC Bioinform. 2007;8:4. doi: 10.1186/1471-2105-8-4 17207271 PMC1780059

[61] Hallgren J, Tsirigos KD, Pedersen MD, Almagro Armenteros JJ, Marcatili P, Nielsen H, et al. DeepTMHMM predicts alpha and beta transmembrane proteins using deep neural networks. Cold Spring Harbor Laboratory; 2022. doi: 10.1101/2022.04.08.487609

[62] Paysan-Lafosse T, Blum M, Chuguransky S, Grego T, Pinto BL, Salazar GA, et al. InterPro in 2022. Nucleic Acids Res. 2023;51(D1):D418–27. doi: 10.1093/nar/gkac993 36350672 PMC9825450

[63] Altschul SF, Koonin EV. Iterated profile searches with PSI-BLAST--a tool for discovery in protein databases. Trends Biochem Sci. 1998;23(11):444–7. doi: 10.1016/s0968-0004(98)01298-5 9852764

[64] Altschul SF, Madden TL, Schäffer AA, Zhang J, Zhang Z, Miller W, et al. Gapped BLAST and PSI-BLAST: a new generation of protein database search programs. Nucleic Acids Res. 1997;25(17):3389–402. doi: 10.1093/nar/25.17.3389 9254694 PMC146917

[65] Varadi M, Bertoni D, Magana P, Paramval U, Pidruchna I, Radhakrishnan M, et al. AlphaFold Protein Structure Database in 2024: providing structure coverage for over 214 million protein sequences. Nucleic Acids Res. 2024;52(D1):D368–75. doi: 10.1093/nar/gkad1011 37933859 PMC10767828

[66] Jumper J, Evans R, Pritzel A, Green T, Figurnov M, Ronneberger O, et al. Highly accurate protein structure prediction with AlphaFold. Nature. 2021;596(7873):583–9. doi: 10.1038/s41586-021-03819-2 34265844 PMC8371605

[67] Pettersen EF, Goddard TD, Huang CC, Couch GS, Greenblatt DM, Meng EC, et al. UCSF Chimera--a visualization system for exploratory research and analysis. J Comput Chem. 2004;25(13):1605–12. doi: 10.1002/jcc.20084 15264254

[68] Johnson MS, Sutcliffe MJ, Blundell TL. Molecular anatomy: phyletic relationships derived from three-dimensional structures of proteins. J Mol Evol. 1990;30(1):43–59. doi: 10.1007/BF02102452 2107323

[69] Krissinel E, Henrick K. Secondary-structure matching (SSM), a new tool for fast protein structure alignment in three dimensions. Acta Crystallogr D Biol Crystallogr. 2004;60(Pt 12 Pt 1):2256–68. doi: 10.1107/S0907444904026460 15572779

[70] Zhang Y, Skolnick J. TM-align: a protein structure alignment algorithm based on the TM-score. Nucleic Acids Res. 2005;33(7):2302–9. doi: 10.1093/nar/gki524 15849316 PMC1084323

[71] Kim Y, Ponomarenko J, Zhu Z, Tamang D, Wang P, Greenbaum J, et al. Immune epitope database analysis resource. Nucleic Acids Res. 2012;40(Web Server issue):W525-30. doi: 10.1093/nar/gks438 22610854 PMC3394288

[72] Andreatta M, Nielsen M. Gapped sequence alignment using artificial neural networks: application to the MHC class I system. Bioinformatics. 2016;32(4):511–7. doi: 10.1093/bioinformatics/btv639 26515819 PMC6402319

[73] Larsen MV, Lundegaard C, Lamberth K, Buus S, Lund O, Nielsen M. Large-scale validation of methods for cytotoxic T-lymphocyte epitope prediction. BMC Bioinform. 2007;8:424. doi: 10.1186/1471-2105-8-424 17973982 PMC2194739

[74] Lund O, Nielsen M, Kesmir C, Petersen AG, Lundegaard C, Worning P, et al. Definition of supertypes for HLA molecules using clustering of specificity matrices. Immunogenetics. 2004;55(12):797–810. doi: 10.1007/s00251-004-0647-4 14963618

[75] Calis JJA, Maybeno M, Greenbaum JA, Weiskopf D, De Silva AD, Sette A, et al. Properties of MHC class I presented peptides that enhance immunogenicity. PLoS Comput Biol. 2013;9(10):e1003266. doi: 10.1371/journal.pcbi.1003266 24204222 PMC3808449

[76] Moise L, McMurry JA, Buus S, Frey S, Martin WD, De Groot AS. In silico-accelerated identification of conserved and immunogenic variola/vaccinia T-cell epitopes. Vaccine. 2009;27(46):6471–9. doi: 10.1016/j.vaccine.2009.06.018 19559119 PMC2838212

[77] Wang P, Sidney J, Kim Y, Sette A, Lund O, Nielsen M, et al. Peptide binding predictions for HLA DR, DP and DQ molecules. BMC Bioinform. 2010;11:568. doi: 10.1186/1471-2105-11-568 21092157 PMC2998531

[78] Wang P, Sidney J, Dow C, Mothé B, Sette A, Peters B. A systematic assessment of MHC class II peptide binding predictions and evaluation of a consensus approach. PLoS Comput Biol. 2008;4(4):e1000048. doi: 10.1371/journal.pcbi.1000048 18389056 PMC2267221

[79] Chauhan V, Rungta T, Goyal K, Singh MP. Designing a multi-epitope based vaccine to combat Kaposi Sarcoma utilizing immunoinformatics approach. Sci Rep. 2019;9(1):2517. doi: 10.1038/s41598-019-39299-8 30792446 PMC6385272

[80] Gupta S, Kapoor P, Chaudhary K, Gautam A, Kumar R, Open Source Drug Discovery Consortium, et al. In silico approach for predicting toxicity of peptides and proteins. PLoS One. 2013;8(9):e73957. doi: 10.1371/journal.pone.0073957 24058508 PMC3772798

[81] Gupta S, Kapoor P, Chaudhary K, Gautam A, Kumar R, Raghava GPS. Peptide toxicity prediction. Methods Mol Biol. 2015;1268:143–57. doi: 10.1007/978-1-4939-2285-7_7 25555724

[82] Binder RJ. Functions of heat shock proteins in pathways of the innate and adaptive immune system. J Immunol. 2014;193(12):5765–71. doi: 10.4049/jimmunol.1401417 25480955 PMC4304677

[83] Dhanda SK, Vir P, Raghava GPS. Designing of interferon-gamma inducing MHC class-II binders. Biol Direct. 2013;8:30. doi: 10.1186/1745-6150-8-30 24304645 PMC4235049

[84] Shen Y, Maupetit J, Derreumaux P, Tufféry P. Improved PEP-FOLD approach for peptide and miniprotein structure prediction. J Chem Theory Comput. 2014;10(10):4745–58. doi: 10.1021/ct500592m 26588162

[85] Lamiable A, Thévenet P, Rey J, Vavrusa M, Derreumaux P, Tufféry P. PEP-FOLD3: faster de novo structure prediction for linear peptides in solution and in complex. Nucleic Acids Res. 2016;44(W1):W449-54. doi: 10.1093/nar/gkw329 27131374 PMC4987898

[86] Macdonald WA, Chen Z, Gras S, Archbold JK, Tynan FE, Clements CS, et al. T cell allorecognition via molecular mimicry. Immunity. 2009;31(6):897–908. doi: 10.1016/j.immuni.2009.09.025 20064448

[87] Toor JS, Rao AA, McShan AC, Yarmarkovich M, Nerli S, Yamaguchi K, et al. A recurrent mutation in anaplastic lymphoma kinase with distinct neoepitope conformations. Front Immunol. 2018;9:99. doi: 10.3389/fimmu.2018.00099 29441070 PMC5797543

[88] Anjanappa R, Garcia-Alai M, Kopicki J-D, Lockhauserbäumer J, Aboelmagd M, Hinrichs J, et al. Structures of peptide-free and partially loaded MHC class I molecules reveal mechanisms of peptide selection. Nat Commun. 2020;11(1):1314. doi: 10.1038/s41467-020-14862-4 32161266 PMC7066147

[89] Kumar P, Vahedi-Faridi A, Saenger W, Merino E, López de Castro JA, Uchanska-Ziegler B, et al. Structural basis for T cell alloreactivity among three HLA-B14 and HLA-B27 antigens. J Biol Chem. 2009;284(43):29784–97. doi: 10.1074/jbc.M109.038497 19617632 PMC2785609

[90] Scally SW, Petersen J, Law SC, Dudek NL, Nel HJ, Loh KL, et al. A molecular basis for the association of the HLA-DRB1 locus, citrullination, and rheumatoid arthritis. J Exp Med. 2013;210(12):2569–82. doi: 10.1084/jem.20131241 24190431 PMC3832918

[91] Petersen J, Ciacchi L, Tran MT, Loh KL, Kooy-Winkelaar Y, Croft NP, et al. T cell receptor cross-reactivity between gliadin and bacterial peptides in celiac disease. Nat Struct Mol Biol. 2020;27(1):49–61. doi: 10.1038/s41594-019-0353-4 31873306

[92] Abualrous ET, Stolzenberg S, Sticht J, Wieczorek M, Roske Y, Günther M, et al. MHC-II dynamics are maintained in HLA-DR allotypes to ensure catalyzed peptide exchange. Nat Chem Biol. 2023;19(10):1196–204. doi: 10.1038/s41589-023-01316-3 37142807 PMC10522485

[93] Galperin M, Farenc C, Mukhopadhyay M, Jayasinghe D, Decroos A, Benati D, et al. CD4+ T cell-mediated HLA class II cross-restriction in HIV controllers. Sci Immunol. 2018;3(24):eaat0687. doi: 10.1126/sciimmunol.aat0687 29884618

[94] Esquivel-Rodriguez J, Filos-Gonzalez V, Li B, Kihara D. Pairwise and multimeric protein-protein docking using the LZerD program suite. Methods Mol Biol. 2014;1137:209–34. doi: 10.1007/978-1-4939-0366-5_15 24573484

[95] Jiménez J, Doerr S, Martínez-Rosell G, Rose AS, De Fabritiis G. DeepSite: protein-binding site predictor using 3D-convolutional neural networks. Bioinformatics. 2017;33(19):3036–42. doi: 10.1093/bioinformatics/btx350 28575181

[96] Thakur M, Bateman A, Brooksbank C, Freeberg M, Harrison M, Hartley M, et al. EMBL’s European Bioinformatics Institute (EMBL-EBI) in 2022. Nucleic Acids Res. 2023;51(D1):D9–17. doi: 10.1093/nar/gkac1098 36477213 PMC9825486

[97] Jo S, Kim T, Iyer VG, Im W. CHARMM-GUI: a web-based graphical user interface for CHARMM. J Comput Chem. 2008;29(11):1859–65. doi: 10.1002/jcc.20945 18351591

[98] Brooks BR, Brooks CL 3rd, Mackerell AD Jr, Nilsson L, Petrella RJ, Roux B, et al. CHARMM: the biomolecular simulation program. J Comput Chem. 2009;30(10):1545–614. doi: 10.1002/jcc.21287 19444816 PMC2810661

[99] Lee J, Cheng X, Swails JM, Yeom MS, Eastman PK, Lemkul JA, et al. CHARMM-GUI input generator for NAMD, GROMACS, AMBER, OpenMM, and CHARMM/OpenMM simulations using the CHARMM36 additive force field. J Chem Theory Comput. 2016;12(1):405–13. doi: 10.1021/acs.jctc.5b00935 26631602 PMC4712441

[100] Darden T, York D, Pedersen L. Particle mesh Ewald: An N⋅log(N) method for Ewald sums in large systems. J Chem Phys. 1993;98(12):10089–92. doi: 10.1063/1.464397

[101] Huang J, Rauscher S, Nawrocki G, Ran T, Feig M, de Groot BL, et al. CHARMM36m: an improved force field for folded and intrinsically disordered proteins. Nat Methods. 2017;14(1):71–3. doi: 10.1038/nmeth.4067 27819658 PMC5199616

[102] Parrinello M, Rahman A. Polymorphic transitions in single crystals: A new molecular dynamics method. J Appl Phys. 1981;52(12):7182–90. doi: 10.1063/1.328693

[103] Lindahl A, Hess van der S. GROMACS 2020.4 manual. 2020. 10.5281/zenodo.4054996

[104] Laskowski RA, Swindells MB. LigPlot+: multiple ligand-protein interaction diagrams for drug discovery. J Chem Inf Model. 2011;51(10):2778–86. doi: 10.1021/ci200227u 21919503

[105] Kern I, Schoffer O, Kiess W, Henker J, Laaß MW, Winkler U, et al. Incidence trends of pediatric onset inflammatory bowel disease in the years 2000-2009 in Saxony, Germany-first results of the Saxon Pediatric IBD registry. PLoS One. 2021;16(1):e0243774. doi: 10.1371/journal.pone.0243774 33395450 PMC7781364

[106] Kugathasan S, Denson LA, Walters TD, Kim M-O, Marigorta UM, Schirmer M, et al. Prediction of complicated disease course for children newly diagnosed with Crohn’s disease: a multicentre inception cohort study. Lancet. 2017;389(10080):1710–8. doi: 10.1016/S0140-6736(17)30317-3 28259484 PMC5719489

[107] Love MI, Huber W, Anders S. Moderated estimation of fold change and dispersion for RNA-seq data with DESeq2. Genome Biol. 2014;15(12):550. doi: 10.1186/s13059-014-0550-8 25516281 PMC4302049

[108] Langmead B, Salzberg SL. Fast gapped-read alignment with Bowtie 2. Nat Methods. 2012;9(4):357–9. doi: 10.1038/nmeth.1923 22388286 PMC3322381

[109] Li J, Jia H, Cai X, Zhong H, Feng Q, Sunagawa S, et al. An integrated catalog of reference genes in the human gut microbiome. Nat Biotechnol. 2014;32(8):834–41. doi: 10.1038/nbt.2942 24997786

[110] Norouzi-Beirami MH, Marashi S-A, Banaei-Moghaddam AM, Kavousi K. Beyond taxonomic analysis of microbiomes: a functional approach for revisiting microbiome changes in colorectal cancer. Front Microbiol. 2020;10:3117. doi: 10.3389/fmicb.2019.03117 32038558 PMC6990412

[111] James V. Miranda L. PySwarms: a research toolkit for Particle Swarm optimization in python. JOSS. 2018;3(21):433. doi: 10.21105/joss.00433

[112] Lu G, Hao X, Chen W-H, Mu S. GAAD: a gene and autoimmiune disease association database. Gen Proteomics Bioinform. 2018;16(4):252–61. doi: 10.1016/j.gpb.2018.05.001 30268934 PMC6205079

[113] Piñero J, Ramírez-Anguita JM, Saüch-Pitarch J, Ronzano F, Centeno E, Sanz F, et al. The DisGeNET knowledge platform for disease genomics: 2019 update. Nucleic Acids Res. 2020;48(D1):D845–55. doi: 10.1093/nar/gkz1021 31680165 PMC7145631

[114] Cariaso M, Lennon G. SNPedia: a wiki supporting personal genome annotation, interpretation and analysis. Nucleic Acids Res. 2012;40(Database issue):D1308–12. doi: 10.1093/nar/gkr798 22140107 PMC3245045

[115] Martin FJ, Amode MR, Aneja A, Austine-Orimoloye O, Azov AG, Barnes I, et al. Ensembl 2023. Nucleic Acids Res. 2023;51(D1):D933–41. doi: 10.1093/nar/gkac958 36318249 PMC9825606

[116] Amberger JS, Bocchini CA, Scott AF, Hamosh A. OMIM.org: leveraging knowledge across phenotype-gene relationships. Nucleic Acids Res. 2019;47(D1):D1038–43. doi: 10.1093/nar/gky1151 30445645 PMC6323937

[117] Kopanos C, Tsiolkas V, Kouris A, Chapple CE, Albarca Aguilera M, Meyer R, et al. VarSome: the human genomic variant search engine. Bioinformatics. 2019;35(11):1978–80. doi: 10.1093/bioinformatics/bty897 30376034 PMC6546127

[118] numpy.corrcoef — NumPy v2.0 manual. [Accessed 2024 July 30]. https://numpy.org/doc/stable/reference/generated/numpy.corrcoef.html

[119] Harris CR, Millman KJ, van der Walt SJ, Gommers R, Virtanen P, Cournapeau D, et al. Array programming with NumPy. Nature. 2020;585(7825):357–62. doi: 10.1038/s41586-020-2649-2 32939066 PMC7759461

[120] Kanehisa M, Goto S. KEGG: kyoto encyclopedia of genes and genomes. Nucleic Acids Res. 2000;28(1):27–30. doi: 10.1093/nar/28.1.27 10592173 PMC102409

[121] Norouzi-Beirami MH, Marashi S-A, Banaei-Moghaddam AM, Kavousi K. CAMAMED: a pipeline for composition-aware mapping-based analysis of metagenomic data. NAR Genom Bioinform. 2021;3(1):lqaa107. doi: 10.1093/nargab/lqaa107 33575649 PMC7787360

[122] Armah-Sekum RE, Szedmak S, Rousu J. Protein function prediction through multi-view multi-label latent tensor reconstruction. BMC Bioinform. 2024;25(1):174. doi: 10.1186/s12859-024-05789-4 38698340 PMC11067221

[123] Madsen P, Isaksen TJ, Siupka P, Tóth AE, Nyegaard M, Gustafsen C, et al. HSPA12A targets the cytoplasmic domain and affects the trafficking of the Amyloid Precursor Protein receptor SorLA. Sci Rep. 2019;9(1):611. doi: 10.1038/s41598-018-37336-6 30679749 PMC6345817

[124] Guan Y, Zhu X, Liang J, Wei M, Huang S, Pan X. Upregulation of HSPA1A/HSPA1B/HSPA7 and downregulation of HSPA9 were related to poor survival in colon cancer. Front Oncol. 2021;11:749673. doi: 10.3389/fonc.2021.749673 34765552 PMC8576338

[125] Wang J, Lee J, Liem D, Ping P. HSPA5 gene encoding Hsp70 chaperone BiP in the endoplasmic reticulum. Gene. 2017;618:14–23. doi: 10.1016/j.gene.2017.03.005 28286085 PMC5632570

[126] Dores-Silva PR, Cauvi DM, Coto ALS, Kiraly VTR, Borges JC, De Maio A. Interaction of HSPA5 (Grp78, BIP) with negatively charged phospholipid membranes via oligomerization involving the N-terminal end domain. Cell Stress Chaperones. 2020;25(6):979–91. doi: 10.1007/s12192-020-01134-9 32725381 PMC7385938

[127] Rehati A, Abuduaini B, Liang Z, Chen D, He F. Identification of heat shock protein family A member 5 (HSPA5) targets involved in nonalcoholic fatty liver disease. Genes Immun. 2023;24(3):124–9. doi: 10.1038/s41435-023-00205-y 37156995 PMC10266971

[128] Kanehisa M, Goto S, Kawashima S, Nakaya A. The KEGG databases at GenomeNet. Nucleic Acids Res. 2002;30(1):42–6. doi: 10.1093/nar/30.1.42 11752249 PMC99091

[129] Kanehisa M, Sato Y, Kawashima M, Furumichi M, Tanabe M. KEGG as a reference resource for gene and protein annotation. Nucleic Acids Res. 2016;44(D1):D457-62. doi: 10.1093/nar/gkv1070 26476454 PMC4702792

[130] Robinson J, Guethlein LA, Cereb N, Yang SY, Norman PJ, Marsh SGE, et al. Distinguishing functional polymorphism from random variation in the sequences of >10,000 HLA-A, -B and -C alleles. PLoS Genet. 2017;13(6):e1006862. doi: 10.1371/journal.pgen.1006862 28650991 PMC5507469

[131] Casalegno Garduño R, Däbritz J. New insights on CD8+ T cells in inflammatory bowel disease and therapeutic approaches. Front Immunol. 2021;12:738762. doi: 10.3389/fimmu.2021.738762 34707610 PMC8542854

[132] Cao SS. Cellular stress responses and gut microbiota in inflammatory bowel disease. Gastroenterol Res Pract. 2018;2018:7192646. doi: 10.1155/2018/7192646 30026758 PMC6031203

[133] Romero-López JP, Domínguez-López ML, Burgos-Vargas R, García-Latorre E. Stress proteins in the pathogenesis of spondyloarthritis. Rheumatol Int. 2019;39(4):595–604. doi: 10.1007/s00296-018-4070-9 29855675

[134] Zhang W, Wei Y, Zhang H, Liu J, Zong Z, Liu Z, et al. Structural alternation in heat shock proteins of activated macrophages. Cells. 2021;10(12):3507. doi: 10.3390/cells10123507 34944015 PMC8700196

[135] Corigliano MG, Sander VA, Sánchez López EF, Ramos Duarte VA, Mendoza Morales LF, Angel SO, et al. Heat shock proteins 90 kDa: immunomodulators and adjuvants in vaccine design against infectious diseases. Front Bioeng Biotechnol. 2021;8:622186. doi: 10.3389/fbioe.2020.622186 33553125 PMC7855457

[136] Nakamura M, Notsu K, Nakagawa M. Heat shock protein 60 negatively regulates the biological functions of ubiquitin-like protein MNSFβ in macrophages. Mol Cell Biochem. 2019;456(1–2):29–39. doi: 10.1007/s11010-018-3487-5 30710197

[137] Murshid A, Gong J, Calderwood SK. The role of heat shock proteins in antigen cross presentation. Front Immunol. 2012;3:63. doi: 10.3389/fimmu.2012.00063 22566944 PMC3342350

[138] Taha EA, Ono K, Eguchi T. Roles of extracellular HSPs as biomarkers in immune surveillance and immune evasion. Int J Mol Sci. 2019;20(18):4588. doi: 10.3390/ijms20184588 31533245 PMC6770223

[139] Martine P, Rébé C. Heat Shock Proteins and inflammasomes. Int J Mol Sci. 2019;20(18):4508. doi: 10.3390/ijms20184508 31547225 PMC6771073

[140] Fu J, Wu H. Structural mechanisms of NLRP3 inflammasome assembly and activation. Annu Rev Immunol. 2023;41:301–16. doi: 10.1146/annurev-immunol-081022-021207 36750315 PMC10159982

[141] El Hadad J, Schreiner P, Vavricka SR, Greuter T. The genetics of inflammatory bowel disease. Mol Diagn Ther. 2024;28(1):27–35. doi: 10.1007/s40291-023-00678-7 37847439 PMC10787003

[142] Nell S, Suerbaum S, Josenhans C. The impact of the microbiota on the pathogenesis of IBD: lessons from mouse infection models. Nat Rev Microbiol. 2010;8(8):564–77. doi: 10.1038/nrmicro2403 20622892

[143] Fridkis-Hareli M. Design of peptide immunotherapies for MHC Class-II-associated autoimmune disorders. Clin Dev Immunol. 2013;2013:826191. doi: 10.1155/2013/826191 24324511 PMC3845387

[144] Dendrou CA, Petersen J, Rossjohn J, Fugger L. HLA variation and disease. Nat Rev Immunol. 2018;18(5):325–39. doi: 10.1038/nri.2017.143 29292391

[145] Birnbaum ME, Mendoza JL, Sethi DK, Dong S, Glanville J, Dobbins J, et al. Deconstructing the peptide-MHC specificity of T cell recognition. Cell. 2014;157(5):1073–87. doi: 10.1016/j.cell.2014.03.047 24855945 PMC4071348

[146] Baker BM, Scott DR, Blevins SJ, Hawse WF. Structural and dynamic control of T-cell receptor specificity, cross-reactivity, and binding mechanism. Immunol Rev. 2012;250(1):10–31. doi: 10.1111/j.1600-065X.2012.01165.x 23046120

[147] Yin Y, Li Y, Mariuzza RA. Structural basis for self-recognition by autoimmune T-cell receptors. Immunol Rev. 2012;250(1):32–48. doi: 10.1111/imr.12002 23046121

[148] Efthymiou G, Sakkas LI, Bogdanos DP. Anti-human Hsp60 autoantibodies in autoimmune and inflammatory rheumatic diseases. In: Asea AAA, Kaur P, eds. Heat Shock Proteins. Cham: Springer International Publishing; 2019: 147–66. doi: 10.1007/978-3-030-23154-5_11

[149] Doody ADH, Kovalchin JT, Mihalyo MA, Hagymasi AT, Drake CG, Adler AJ. Glycoprotein 96 can chaperone both MHC class I- and class II-restricted epitopes for in vivo presentation, but selectively primes CD8+ T cell effector function. J Immunol. 2004;172(10):6087–92. doi: 10.4049/jimmunol.172.10.6087 15128793 PMC2846363

[150] Glanville J, Huang H, Nau A, Hatton O, Wagar LE, Rubelt F, et al. Identifying specificity groups in the T cell receptor repertoire. Nature. 2017;547(7661):94–8. doi: 10.1038/nature22976 28636589 PMC5794212

[151] Tavakoli P, Vollmer-Conna U, Hadzi-Pavlovic D, Grimm MC. A review of inflammatory bowel disease: a model of microbial, immune and neuropsychological integration. Public Health Rev. 2021;42:1603990. doi: 10.3389/phrs.2021.1603990 34692176 PMC8386758

[152] Frank DN, St Amand AL, Feldman RA, Boedeker EC, Harpaz N, Pace NR. Molecular-phylogenetic characterization of microbial community imbalances in human inflammatory bowel diseases. Proc Natl Acad Sci U S A. 2007;104(34):13780–5. doi: 10.1073/pnas.0706625104 17699621 PMC1959459

[153] van Eden W. Immune tolerance therapies for autoimmune diseases based on heat shock protein T-cell epitopes. Philos Trans R Soc Lond B Biol Sci. 2018;373(1738):20160531. doi: 10.1098/rstb.2016.0531 29203716 PMC5717531

[154] Yin D, Li L, Song X, Li H, Wang J, Ju W, et al. A novel multi-epitope recombined protein for diagnosis of human brucellosis. BMC Infect Dis. 2016;16:219. doi: 10.1186/s12879-016-1552-9 27206475 PMC4875615

[155] Singh MK, Shin Y, Han S, Ha J, Tiwari PK, Kim SS, et al. Molecular Chaperonin HSP60: current understanding and future prospects. Int J Mol Sci. 2024;25(10):5483. doi: 10.3390/ijms25105483 38791521 PMC11121636

[156] Scarozza P, Schmitt H, Monteleone G, Neurath MF, Atreya R. Oligonucleotides-A novel promising therapeutic option for IBD. Front Pharmacol. 2019;10:314. doi: 10.3389/fphar.2019.00314 31068803 PMC6491809

[157] Toskas A, Akbar A. IBD therapeutics: what is in the pipeline? Front Gastroenterol. 2022;13(e1):e35–43. doi: 10.1136/flgastro-2022-102130 35812030 PMC9234727

[158] Cai Z, Wang S, Li J. Treatment of inflammatory bowel disease: a comprehensive review. Front Med (Lausanne). 2021;8:765474. doi: 10.3389/fmed.2021.765474 34988090 PMC8720971

[159] Barberá A, Lorenzo N, van Kooten P, van Roon J, de Jager W, Prada D, et al. APL1, an altered peptide ligand derived from human heat-shock protein 60, increases the frequency of Tregs and its suppressive capacity against antigen responding effector CD4 + T cells from rheumatoid arthritis patients. Cell Stress Chaperones. 2016;21(4):735–44. doi: 10.1007/s12192-016-0698-0 27241313 PMC4908004

[160] Candia M, Kratzer B, Pickl WF. On peptides and altered peptide ligands: from origin, mode of action and design to clinical application (Immunotherapy). Int Arch Allergy Immunol. 2016;170(4):211–33. doi: 10.1159/000448756 27642756 PMC7058415

[161] Hoppes R, Oostvogels R, Luimstra JJ, Wals K, Toebes M, Bies L, et al. Altered peptide ligands revisited: vaccine design through chemically modified HLA-A2-restricted T cell epitopes. J Immunol. 2014;193(10):4803–13. doi: 10.4049/jimmunol.1400800 25311806 PMC4226423

[162] Varma S, Faye AS, Kannan A, Lawlor G, Verma A, Axelrad J, et al. Patients with more severe IBD get clostridioides difficile rather than clostridioides difficile increasing the severity of IBD. Dig Dis Sci. 2021;66(9):3113–23. doi: 10.1007/s10620-020-06504-y 32729015

[163] Gupta S, Li D, Ostrov DA, Nguyen CQ. Blocking IAg7 class II major histocompatibility complex by drug-like small molecules alleviated Sjögren’s syndrome in NOD mice. Life Sci. 2022;288:120182. doi: 10.1016/j.lfs.2021.120182 34843735 PMC8883604

[164] Votaw NL, Collier L, Curvino EJ, Wu Y, Fries CN, Ojeda MT, et al. Randomized peptide assemblies for enhancing immune responses to nanomaterials. Biomaterials. 2021;273:120825. doi: 10.1016/j.biomaterials.2021.120825 33901731 PMC8163017

[165] Cruz-Tapias P, Castiblanco J, Anaya J-M. HLA association with autoimmune diseases. El Rosario University Press; 2013. https://www.ncbi.nlm.nih.gov/books/NBK459459/

[166] Farjam M, Zhang G-X, Ciric B, Rostami A. Emerging immunopharmacological targets in multiple sclerosis. J Neurol Sci. 2015;358(1–2):22–30. doi: 10.1016/j.jns.2015.09.346 26440421 PMC5215047

[167] Nardone OM, Zammarchi I, Santacroce G, Ghosh S, Iacucci M. Inflammation-driven colorectal cancer associated with colitis: from pathogenesis to changing therapy. Cancers (Basel). 2023;15(8):2389. doi: 10.3390/cancers15082389 37190315 PMC10136846

[168] Zhang J, Shen L, Johnston SA. Using frameshift peptide arrays for cancer neo-antigens screening. Sci Rep. 2018;8(1):17366. doi: 10.1038/s41598-018-35673-0 30478295 PMC6255861

[169] Lv D, Khawar MB, Liang Z, Gao Y, Sun H. Neoantigens and NK Cells: “Trick or Treat” the cancers? Front Immunol. 2022;13:931862. doi: 10.3389/fimmu.2022.931862 35874694 PMC9302773

[170] Biswas N, Chakrabarti S, Padul V, Jones LD, Ashili S. Designing neoantigen cancer vaccines, trials, and outcomes. Front Immunol. 2023;14:1105420. doi: 10.3389/fimmu.2023.1105420 36845151 PMC9947792

[171] Ho S-Y, Chang C-M, Liao H-N, Chou W-H, Guo C-L, Yen Y, et al. Current trends in neoantigen-based cancer vaccines. Pharmaceuticals (Basel). 2023;16(3):392. doi: 10.3390/ph16030392 36986491 PMC10056833

[172] Shan Y, Lee M, Chang EB. The gut microbiome and inflammatory bowel diseases. Annu Rev Med. 2022;73:455–68. doi: 10.1146/annurev-med-042320-021020 34555295 PMC10012812

[173] Magin WS, Van Kruiningen HJ, Colombel J-F. Immunohistochemical search for viral and bacterial antigens in Crohn’s disease. J Crohns Colitis. 2013;7(2):161–6. doi: 10.1016/j.crohns.2012.03.021 22537638

[174] Eckburg PB, Relman DA. The role of microbes in Crohn’s disease. Clin Infect Dis. 2007;44(2):256–62. doi: 10.1086/510385 17173227

[175] Mirsepasi-Lauridsen HC, Vallance BA, Krogfelt KA, Petersen AM. Escherichia coli pathobionts associated with inflammatory bowel disease. Clin Microbiol Rev. 2019;32(2):e00060-18. doi: 10.1128/CMR.00060-18 30700431 PMC6431131

[176] Ebringer A, Rashid T, Tiwana H, Wilson C. A possible link between Crohn’s disease and ankylosing spondylitis via Klebsiella infections. Clin Rheumatol. 2007;26(3):289–97. doi: 10.1007/s10067-006-0391-2 16941202

[177] Polymeros D, Bogdanos DP, Day R, Arioli D, Vergani D, Forbes A. Does cross-reactivity between mycobacterium avium paratuberculosis and human intestinal antigens characterize Crohn’s disease? Gastroenterology. 2006;131(1):85–96. doi: 10.1053/j.gastro.2006.04.021 16831593

[178] Fang X, Kang L, Qiu Y-F, Li Z-S, Bai Y. Yersinia enterocolitica in Crohn’s disease. Front Cell Infect Microbiol. 2023;13:1129996. doi: 10.3389/fcimb.2023.1129996 36968108 PMC10031030

[179] Triantafillidis JK, Thomaidis T, Papalois A. Terminal ileitis due to yersinia infection: an underdiagnosed situation. Biomed Res Int. 2020;2020:1240626. doi: 10.1155/2020/1240626 32566652 PMC7273408

[180] Lamhonwah A-M, Ackerley C, Onizuka R, Tilups A, Lamhonwah D, Chung C, et al. Epitope shared by functional variant of organic cation/carnitine transporter, OCTN1, Campylobacter jejuni and Mycobacterium paratuberculosis may underlie susceptibility to Crohn’s disease at 5q31. Biochem Biophys Res Commun. 2005;337(4):1165–75. doi: 10.1016/j.bbrc.2005.09.170 16246312

[181] Wang L, Cao Z-M, Zhang L-L, Dai X-C, Liu Z-J, Zeng Y-X, et al. Helicobacter pylori and autoimmune diseases: involving multiple systems. Front Immunol. 2022;13:833424. doi: 10.3389/fimmu.2022.833424 35222423 PMC8866759

[182] Chukwudozie OS, Gray CM, Fagbayi TA, Chukwuanukwu RC, Oyebanji VO, Bankole TT, et al. Immuno-informatics design of a multimeric epitope peptide based vaccine targeting SARS-CoV-2 spike glycoprotein. PLoS One. 2021;16(3):e0248061. doi: 10.1371/journal.pone.0248061 33730022 PMC7968690

[183] Fukaya T, Uto T, Mitoma S, Takagi H, Nishikawa Y, Tominaga M, et al. Gut dysbiosis promotes the breakdown of oral tolerance mediated through dysfunction of mucosal dendritic cells. Cell Rep. 2023;42(5):112431. doi: 10.1016/j.celrep.2023.112431 37099426

[184] Liu C, Su W, Tan Z, Zhang J, Dong W. The interaction between microbiota and immune in intestinal inflammatory diseases: Global research status and trends. Front Cell Infect Microbiol. 2023;13:1128249. doi: 10.3389/fcimb.2023.1128249 36824689 PMC9941562

[185] Shukla HD, Pitha PM. Role of hsp90 in systemic lupus erythematosus and its clinical relevance. Autoimmune Dis. 2012;2012:728605. doi: 10.1155/2012/728605 23091704 PMC3471389

[186] Lu Q, Yang M-F, Liang Y-J, Xu J, Xu H-M, Nie Y-Q, et al. Immunology of inflammatory bowel disease: molecular mechanisms and therapeutics. J Inflamm Res. 2022;15:1825–44. doi: 10.2147/JIR.S353038 35310454 PMC8928114

[187] Qiu X-B, Shao Y-M, Miao S, Wang L. The diversity of the DnaJ/Hsp40 family, the crucial partners for Hsp70 chaperones. Cell Mol Life Sci. 2006;63(22):2560–70. doi: 10.1007/s00018-006-6192-6 16952052 PMC11136209

[188] Stark JL, Mehla K, Chaika N, Acton TB, Xiao R, Singh PK, et al. Structure and function of human DnaJ homologue subfamily a member 1 (DNAJA1) and its relationship to pancreatic cancer. Biochemistry. 2014;53(8):1360–72. doi: 10.1021/bi401329a 24512202 PMC3985919

[189] Cyr DM, Ramos CH. Specification of Hsp70 Function by Hsp40 Co-chaperones. Subcell Biochem. 2023;101:127–39. doi: 10.1007/978-3-031-14740-1_4 36520305

[190] Kampinga HH, Craig EA. The HSP70 chaperone machinery: J proteins as drivers of functional specificity. Nat Rev Mol Cell Biol. 2010;11(8):579–92. doi: 10.1038/nrm2941 20651708 PMC3003299

[191] Zhang X, Luo X, Tian L, Yue P, Li M, Liu K, et al. The gut microbiome dysbiosis and regulation by fecal microbiota transplantation: umbrella review. Front Microbiol. 2023;14:1286429. doi: 10.3389/fmicb.2023.1286429 38029189 PMC10655098

[192] Fiocchi C, Iliopoulos D. Inflammatory bowel disease therapy: beyond the immunome. Front Immunol. 2022;13:864762. doi: 10.3389/fimmu.2022.864762 35615360 PMC9124778

[193] Hou J, Ye X, Feng W, Zhang Q, Han Y, Liu Y, et al. Distance correlation application to gene co-expression network analysis. BMC Bioinformatics. 2022;23(1):81. doi: 10.1186/s12859-022-04609-x 35193539 PMC8862277

[194] Song L, Langfelder P, Horvath S. Comparison of co-expression measures: mutual information, correlation, and model based indices. BMC Bioinform. 2012;13:328. doi: 10.1186/1471-2105-13-328 23217028 PMC3586947

[195] Kontio JAJ, Rinta-Aho MJ, Sillanpää MJ. Estimating Linear and nonlinear gene coexpression networks by semiparametric neighborhood selection. Genetics. 2020;215(3):597–607. doi: 10.1534/genetics.120.303186 32414870 PMC7337083

[196] Oliveira de Biagi CA Jr, Nociti RP, Brotto DB, Funicheli BO, Cássia Ruy P de, Bianchi Ximenez JP, et al. CeTF: an R/Bioconductor package for transcription factor co-expression networks using regulatory impact factors (RIF) and partial correlation and information (PCIT) analysis. BMC Gen. 2021;22(1):624. doi: 10.1186/s12864-021-07918-2 34416858 PMC8379792

[197] Daub CO, Steuer R, Selbig J, Kloska S. Estimating mutual information using B-spline functions--an improved similarity measure for analysing gene expression data. BMC Bioinform. 2004;5:118. doi: 10.1186/1471-2105-5-118 15339346 PMC516800

[198] Marciani DJ. Effects of immunomodulators on the response induced by vaccines against autoimmune diseases. Autoimmunity. 2017;50(7):393–402. doi: 10.1080/08916934.2017.1373766 28906131

[199] Chountoulesi M, Demetzos C. Promising nanotechnology approaches in treatment of autoimmune diseases of central nervous system. Brain Sci. 2020;10(6):338. doi: 10.3390/brainsci10060338 32498357 PMC7349417

[200] Tukaj S, Węgrzyn G. Anti-Hsp90 therapy in autoimmune and inflammatory diseases: a review of preclinical studies. Cell Stress Chaperones. 2016;21(2):213–8. doi: 10.1007/s12192-016-0670-z 26786410 PMC4786535

[201] Orthmann-Murphy JL, Calabresi PA. Therapeutic application of monoclonal antibodies in multiple sclerosis. Clin Pharmacol Ther. 2017;101(1):52–64. doi: 10.1002/cpt.547 27804128

[202] Mao X, Ma J, Jiao C, Tang N, Zhao X, Wang D, et al. Faecalibacterium prausnitzii attenuates DSS-induced colitis by inhibiting the colonization and pathogenicity of Candida albicans. Mol Nutr Food Res. 2021;65:e2100433. doi: 10.1002/mnfr.202100433 34558816

[203] Bezzio C, Della Corte C, Vernero M, Di Luna I, Manes G, Saibeni S. Inflammatory bowel disease and immune-mediated inflammatory diseases: looking at the less frequent associations. Therap Adv Gastroenterol. 2022;15:17562848221115312. doi: 10.1177/17562848221115312 35924080 PMC9340394

[204] Ferrè L, Filippi M, Esposito F. Involvement of genetic factors in multiple sclerosis. Front Cell Neurosci. 2020;14:612953. doi: 10.3389/fncel.2020.612953 33335478 PMC7735985

[205] Lucafò M, Curci D, Franzin M, Decorti G, Stocco G. Inflammatory bowel disease and risk of colorectal cancer: an overview from pathophysiology to pharmacological prevention. Front Pharmacol. 2021;12:772101. doi: 10.3389/fphar.2021.772101 34744751 PMC8563785

[206] Frigerio S, Lartey DA, D’Haens GR, Grootjans J. The role of the immune system in IBD-associated colorectal cancer: from pro to anti-tumorigenic mechanisms. Int J Mol Sci. 2021;22(23):12739. doi: 10.3390/ijms222312739 34884543 PMC8657929

[207] Epistola R, Do T, Vankina R, Wu D, Yeh J, Fleischman MW, et al. Immune Thrombocytopenic Purpura (ITP) as an uncommon extraintestinal complication of crohn’s disease: case vignette and systematic literature review. Case Rep Hematol. 2020;2020:4785759. doi: 10.1155/2020/4785759 32274225 PMC7136770

[208] Wang F, Xie J, Xiong H, Xie Y. A bibliometric analysis of inflammatory bowel disease and COVID-19 researches. Front Public Health. 2023;11:1039782. doi: 10.3389/fpubh.2023.1039782 36794064 PMC9922853

[209] Baishya J, Bisht K, Rimbey JN, Yihunie KD, Islam S, Al Mahmud H, et al. The impact of intraspecies and interspecies bacterial interactions on disease outcome. Pathogens. 2021;10(2):96. doi: 10.3390/pathogens10020096 33494265 PMC7909810

[210] Schirmer M, Garner A, Vlamakis H, Xavier RJ. Microbial genes and pathways in inflammatory bowel disease. Nat Rev Microbiol. 2019;17: 497–511. doi: 10.1038/s41579-019-0213-6 31249397 PMC6759048

[211] Lavelle A, Sokol H. Gut microbiota: Beyond metagenomics, metatranscriptomics illuminates microbiome functionality in IBD. Nat Rev Gastroenterol Hepatol. 2018;15(4):193–4. doi: 10.1038/nrgastro.2018.15 29463904

[212] Xu X, Ocansey DKW, Hang S, Wang B, Amoah S, Yi C, et al. The gut metagenomics and metabolomics signature in patients with inflammatory bowel disease. Gut Pathog. 2022;14(1):26. doi: 10.1186/s13099-022-00499-9 35729658 PMC9215062

[213] Massimino L, Lamparelli LA, Houshyar Y, D’Alessio S, Peyrin-Biroulet L, Vetrano S, et al. The inflammatory bowel disease transcriptome and metatranscriptome meta-analysis (IBD TaMMA) framework. Nat Comput Sci. 2021;1(8):511–5. doi: 10.1038/s43588-021-00114-y 38217242 PMC10766544

[214] Zhou H, Beltrán JF, Brito IL. Host-microbiome protein-protein interactions capture disease-relevant pathways. Genome Biol. 2022;23(1):72. doi: 10.1186/s13059-022-02643-9 35246229 PMC8895870

[215] Poluri KM, Gulati K, Tripathi DK, Nagar N. Protein–Protein Interactions in Immune Disorders and Inflammation. In: Poluri KM, Gulati K, Tripathi DK, Nagar N, eds. Protein-Protein Interactions. Springer Nature Singapore; 2023: 171–206. doi: 10.1007/978-981-99-2423-3_4

[216] Guan Q. A comprehensive review and update on the pathogenesis of inflammatory bowel disease. J Immunol Res. 2019;2019:7247238. doi: 10.1155/2019/7247238 31886308 PMC6914932

[217] Hentschel V, Klaus J. Molecular medicine-based IBD treatment strategies—we take it personally! Front Gastroenterol. 2023;2. doi: 10.3389/fgstr.2023.1226048 PMC12952339 41821787

[218] Chen J, Xu F, Ruan X, Sun J, Zhang Y, Zhang H, et al. Therapeutic targets for inflammatory bowel disease: proteome-wide Mendelian randomization and colocalization analyses. EBioMedicine. 2023;89:104494. doi: 10.1016/j.ebiom.2023.104494 36857861 PMC9986512

